# Functional genetic variation in *pe*/*ppe* genes contributes to diversity in *Mycobacterium tuberculosis* lineages and potential interactions with the human host

**DOI:** 10.3389/fmicb.2023.1244319

**Published:** 2023-10-09

**Authors:** Paula Josefina Gómez-González, Anna D. Grabowska, Leopold D. Tientcheu, Anthony G. Tsolaki, Martin L. Hibberd, Susana Campino, Jody E. Phelan, Taane G. Clark

**Affiliations:** ^1^Faculty of Infectious and Tropical Diseases, London School of Hygiene and Tropical Medicine, London, United Kingdom; ^2^Department of Biophysics, Physiology and Pathophysiology, Medical University of Warsaw, Warsaw, Poland; ^3^MRC Unit, The Gambia at the London School of Hygiene and Tropical Medicine, Vaccines and Immunity Theme, Fajara, The Gambia; ^4^Department of Life Sciences, Brunel University London, Uxbridge, United Kingdom; ^5^Faculty of Epidemiology and Population Health, London School of Hygiene and Tropical Medicine, London, United Kingdom

**Keywords:** *Mycobacerium tuberculosis*, genomics, MTBC, diversity, *pe/ppe* family of genes

## Abstract

**Introduction:**

Around 10% of the coding potential of *Mycobacterium tuberculosis*is constituted by two poorly understood gene families, the *pe* and *ppe* loci, thought to be involved in host-pathogen interactions. Their repetitive nature and high GC content have hindered sequence analysis, leading to exclusion from whole-genome studies. Understanding the genetic diversity of *pe/ppe* families is essential to facilitate their potential translation into tools for tuberculosis prevention and treatment.

**Methods:**

To investigate the genetic diversity of the 169 *pe*/*ppe* genes, we performed a sequence analysis across 73 long-read assemblies representing seven different lineages of *M. tuberculosis* and *M. bovis* BCG. Individual *pe/ppe* gene alignments were extracted and diversity and conservation across the different lineages studied.

**Results:**

The *pe*/*ppe* genes were classified into three groups based on the level of protein sequence conservation relative to H37Rv, finding that >50% were conserved, with indels in *pe_pgrs* and *ppe_mptr* sub-families being major drivers of structural variation. Gene rearrangements, such as duplications and gene fusions, were observed between *pe* and *pe_pgrs* genes. Inter-lineage diversity revealed lineage-specific SNPs and indels.

**Discussion:**

The high level of *pe/ppe* genes conservation, together with the lineage-specific findings, suggest their phylogenetic informativeness. However, structural variants and gene rearrangements differing from the reference were also identified, with potential implications for pathogenicity. Overall, improving our knowledge of these complex gene families may have insights into pathogenicity and inform the development of much-needed tools for tuberculosis control.

## 1. Introduction

Tuberculosis (TB) disease, caused by bacteria of the *Mycobacterium tuberculosis* complex (MTBC), is a major global public health problem with drug resistance making its control difficult (World Health Organization, 2021). The available vaccine, Bacillus Calmette-Guérin (BCG), has limited efficacy and recent attempts to develop more productive vaccines have been unsuccessful, in part due to the insufficient understanding of host-pathogen interactions (Sable et al., 2019). The MTBC genome has a low overall genetic diversity and a striking clonal population structure, with nine lineages (L1-L9), which are postulated to have different impacts on pathogenesis, disease diagnosis, treatment outcome and vaccine efficacy (Coscolla and Gagneux, 2014; Tientcheu et al., 2016, 2017). Of the nine phylogeographic lineages identified, three are referred to as evolutionarily “ancient” (L1, L5, L6), and three “modern” (L2, L3, and L4). While some genetic differences between lineages have been identified (Napier et al., 2020), the molecular mechanisms responsible for differences in pathogenesis and virulence remain largely unknown.

The H37Rv *M. tuberculosis* (*Mtb*) genome has unique *pe* (*n* = 100) and *ppe* (*n* = 69) genes, which are found in larger numbers in pathogenic mycobacteria compared to saprophytic or avirulent species (Gey van Pittius et al., 2006; Akhter et al., 2012; McGuire et al., 2012), and therefore suggested to play a role in pathogenicity and virulence. These two families constitute ~10% of the *Mtb* coding potential and have a conserved N-terminal domain, within which signature proline-glutamate (PE) and proline-proline-glutamate (PPE) motifs can be identified in most of the protein products (Cole et al., 1998). In contrast, the C-terminal sequences are more variable and of various sizes. Their evolution and expansion have been proposed to be linked to a series of duplication events of the early secreted antigenic target 6 kDa (ESAT-6) gene clusters (Gey van Pittius et al., 2006; Abdallah et al., 2007), together with insertions/deletions (indels) and homologous recombination (Medha and Sharma, 2021). Often, *pe*/*ppe* genes are hotspots of polymorphisms and recombination, showing higher diversity than the rest of the genome, while others are conserved across lineages, implying different functional roles (Talarico et al., 2005, 2008; Karboul et al., 2008; McEvoy et al., 2012; Copin et al., 2014; Phelan et al., 2016).

Despite the function of PE and PPE proteins being poorly understood, some have been demonstrated to have various roles in host-pathogen interactions and immune evasion. Their subcellular localization requires them to be secreted by the ESX system (Ates, 2020), with PPE38 playing an essential role in the secretion of PE_PGRS and PPE_MPTR proteins (Ates et al., 2018). The disruption of *ppe38* observed in Beijing strains (L2) has been associated with a hypervirulent phenotype (Ates et al., 2018), thereby demonstrating how strain-specific structural variants may affect pathogenesis and virulence of different MTBC lineages. The *pe/ppe* proteins are highly immunogenic and, therefore, promising targets for vaccine and diagnostic development (Qian et al., 2020). The apparent polymorphic and repetitive nature of these genes was proposed as a source of antigenic variation (Talarico et al., 2008; Tundup et al., 2008; Akhter et al., 2012); however, highly conserved T-cell epitopes have been found among *pe_pgrs* genes (Copin et al., 2014), which contradict this theory.

To provide a better understanding of the role of *pe*/*ppe* genes in pathogenesis, immune evasion and complement immunogenic assays and evaluations of vaccine candidates, there is a need to fully characterize the genetic diversity across the different MTBC lineages. However, *pe*/*ppe* genes have been systematically excluded from analyzes due to the difficulties in reliably aligning sequences to the high GC repetitive regions (Phelan et al., 2016; Meehan et al., 2019; Ates, 2020). Although the availability of high throughput short sequencing technologies has revolutionized the study of MTBC genetic diversity, an increased number of coverage blind spots in short-read sequencing occurs in *pe*/*ppe* genes (Modlin et al., 2021). This limitation can be overcome by long-read sequencing technologies, such as the PacBio and Oxford Nanopore Technology platforms (Elghraoui et al., 2017). To characterize these elusive genes and genetic variants, we have performed an *in-silico* analysis of the 169 *pe*/*ppe* gene sequences across 73 MTBC isolates with (near-)complete assembled genomes, representing seven different lineages. We have classified the *pe*/*ppe* genes based on their conservation profiles across the MTBC, identifying lineage-specific markers among the conserved genes and lineage patterns responsible for disrupted protein sequences, likely to have functional consequences. Overall, using long read sequence data, we provide the first comprehensive analysis of the genetic diversity among the *pe*/*ppe* families to assist the development of TB control tools.

## 2. Materials and methods

### 2.1. Selection of samples, culture and sequencing

A total of 73 PacBio assemblies were used for the analysis. Ten samples were cultured at LSHTM CL3 laboratories and sequenced for this study, sourced from TB patients in the Karonga district (Malawi) between 2001 and 2009. Briefly, *Mtb* clinical isolates derived from patient’s sputum were cultured to mid-log phase (optical density = 0.6–0.8) in Middlebrook 7H9 supplemented with 0.05% Tween 80 and 10% albumin-dextrose-catalase (ADC) at 37°C in roller bottles. DNA was extracted from passage 2 by heat-inactivation followed by the CTAB-chloroform-isoamyl alcohol method (Somerville et al., 2005). DNA samples were sequenced with single-molecule real-time (SMRT) sequencing technology from Pacific Biosciences (PacBio) RSII through The Applied Genomics Center at LSHTM. To generate genome assemblies, *de novo* methods were performed on the raw sequencing data from the ten isolates together with other 27 samples previously sequenced (Phelan et al., 2018; Gomez-Gonzalez et al., 2019), using Flye software (Kolmogorov et al., 2019). These assembled genomes were base corrected using Illumina short-reads using Pilon software (Walker et al., 2014). The Illumina short-reads from the different samples used for the assembly improvement were publicly available from previous studies (for accession numbers, see Supplementary Table S1). The remaining 35 assembled genomes studied were publicly available and sourced from the ENA (for accession numbers, see Supplementary Table S1). To ensure robust inference, only high-quality assemblies with a maximum of 8 contigs were included in the analysis. Lineage and sub-lineage profiling were performed using TB-Profiler software (Phelan et al., 2019).

### 2.2. Whole-genome population genetics analysis

The H37Rv reference genome (ASM19595v2) was used for the population genetics analysis. Snippy software (Seemann, 2015) was used to simulate reads from assemblies and to call variants (SNPs and indels with a minimum coverage of 10 and a minimum fraction differing from reference of 0.9) at a whole-genome level against the H37Rv reference genome. No regions were excluded from this analysis. The R packages PopGenome (Pfeifer et al., 2014) and SeqinR (Charif and Lobry, 2007) were used for the population genetics analysis. In brief, Nei’s π nucleotide diversity per site (SNP π), indel diversity per site (indel π) and absolute divergence (*dxy*) were calculated in sliding windows throughout the genome for the different populations (e.g., ancient and modern lineages). The average of the three parameters was calculated for the comparison between populations. The *dN*/*dS* pairwise ratios were calculated by concatenating the coding regions relative to the reference H37Rv. Statistical differences in diversity and divergence parameters between gene functional groups were calculated using analysis of variance (ANOVA), where *p*-values were corrected by multiple comparisons using Tukey’s Honest Significant Differences (HSD) test. Functional groups were considered as defined previously (Cole et al., 1998). IQ-TREE software (Nguyen et al., 2015) was used for the phylogenetic reconstruction of maximum-likelihood trees using a GTR + I + G substitution model using SNPs and/or indels alignments of the samples analyzed. The NCBI prokaryotic genome annotation pipeline PGAP (Tatusova et al., 2016) was used to annotate the genomes and validate gene rearrangements. Differences in annotation calls between H37Rv and other isolates were investigated using BLAST searching and matching.

### 2.3. *Pe*/*ppe* gene extraction, alignment and classification

The *pe* and *ppe* gene alignments were generated using a customized pipeline. In brief, non-*pe*/*ppe* flanking genes were found in the assemblies using blast software (Zhang et al., 2000) and used as anchors to extract the sequence, which were subsequently aligned with MAFFT software (Katoh and Standley, 2013). Where flanking genes were in different contigs or could not be mapped to the assemblies, genes were considered missing in samples. Single *pe*/*ppe* gene alignments were obtained relative to the H37Rv sequences and curated manually if necessary. SNPs and indels for each gene were obtained using the H37Rv reference. Levels of disruption that these variants caused on the protein sequence were assigned (0 = no variants or synonymous SNPs; 1 = non-synonymous SNPs; 2 = in-frame indels; 3 = SNPs or frameshifts leading to changes in start/stop codons, deletions of >50% of the coding region, or completely missing or insertions >1,000 bp). To investigate whether individual genes were conserved across the different lineages, each *pe*/*ppe* gene was classified into one of the three classes or categories: conserved (C), structurally non-conserved (S), and unique *k-mer* profile (K) (see Supplementary Figure S1). Briefly, for each gene alignment, if two or more isolates were assigned a value of 3 as described above, the gene was considered structurally non-conserved (class S). In some genes, some samples had a high density of SNPs in some regions while still maintaining the same sequence length as the reference. Other genes had samples that contained completely novel sequences insertions. In an attempt to characterize the presence of these, DSK software (Rizk et al., 2013) was used to count *k-mers*. For each gene alignment, the *k-mer* profile was obtained. Those that did not show structural variants but had enrichments of unique *k-mers* because of SNPs or indels, were considered as class K.

### 2.4. Illumina short-read data analysis

A database of ~30 k isolates (“30 k dataset”) with short-read Illumina data and representing every lineage (L1-L6 and *M/bovis*) was used (Napier et al., 2020). Short-reads were aligned to the reference with BWA-MEM (Li and Durbin, 2009), and the coverage per gene per sample was calculated with BEDTools software (Quinlan and Hall, 2010). The coverage was normalized by four housekeeping genes (*gyrA*, *gyrB*, *rpoB* and *rpoC*) and compared between *pe*/*ppe* genes and the rest of the genome. For the comparison between groups, *pe*/*ppe* genes were divided into the previously explained categories (C, S, K). In some cases, categories were combined if samples sizes were small. Statistical differences in the means between categories were assessed using T-tests.

### 2.5. The *pe* and *ppe* genes sequence analysis

For the population genetics analysis of the individual *pe*/*ppe* genes, the alignments obtained by the previous pipeline were used. Population genetics parameters (nucleotide and indel diversity and divergence) for individual genes were calculated using PopGenome R package (Pfeifer et al., 2014). The BUSTED method was used to calculate *dN*/*dS* ratios (Murrell et al., 2015). Identification of known domains was performed with Pfam software (Mistry et al., 2021). T-tests were applied to calculate the statistical differences for nucleotide and indel diversity between the different domains or gene groups. AlphaFold software (Jumper et al., 2021) was used for the prediction of protein structure models. For all variants identified in *pe*/*ppe* genes, fixation index (*F_ST_*) values were calculated to assess allele differences across lineages. As a validation of variants with *F_ST_* values of 1 (perfect differentiation), allele frequencies in the 30 k dataset were obtained (Napier et al., 2020). For the consideration of lineage-specific variants, an allele frequency of 0 in other lineages and > 0.95 in the corresponding lineage was required.

### 2.6. Data availability

The sequence data supporting the conclusions of this article have been deposited in the ENA (Supplementary Table S1 for accession numbers).

## 3. Results

### 3.1. Genome-wide SNP and indel nucleotide diversity

**Figure 1:**
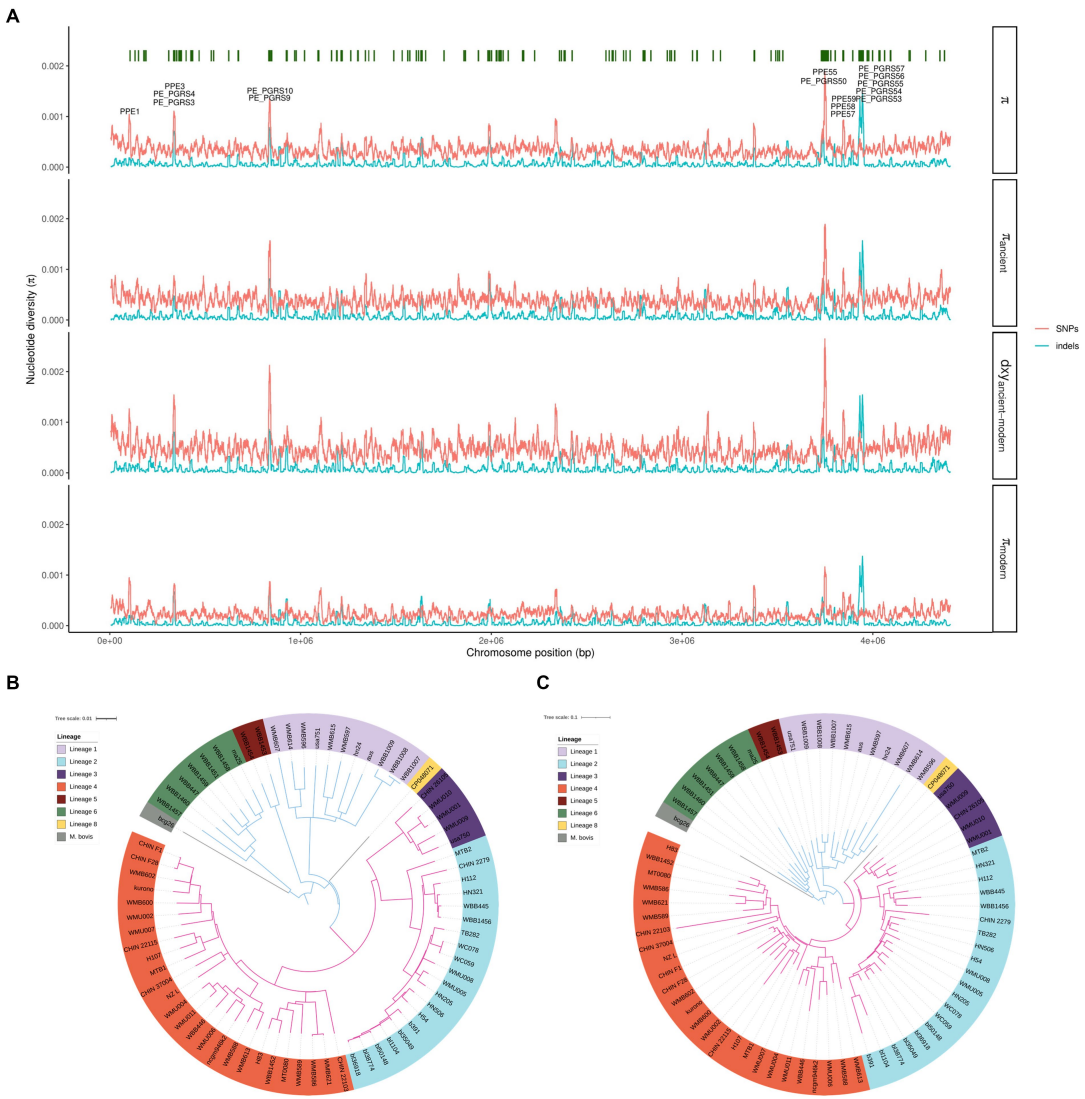
**(A)** Whole genome SNP nucleotide and indel diversity. From top to bottom, the first track shows nucleotide diversity along the chromosome, with the peaks over 0.001 highlighted in a box. The *pe*/*ppe* genes in the peaks of nucleotide diversity are annotated. Green bars show where *pe*/*ppe* genes are located along the genome. The second track shows nucleotide diversity in ancient lineages. The third track shows absolute divergence between ancient and modern lineages. The fourth track shows nucleotide diversity in modern lineages. Line in read represents SNPs diversity and in blue indel diversity. **(B)** Maximum likelihood phylogenetic tree reconstructed with whole genome SNPs (*n* = 20,144). **(C)** Maximum likelihood phylogenetic tree reconstructed with whole genome indels (*n* = 6,632). Ancient lineages are represented in blue, modern lineages in pink.

A total of 73 clinical MTBC isolates with PacBio long-read sequencing data and complete genomes (Phelan et al., 2018; Gomez-Gonzalez et al., 2019) were included in the analysis (Supplementary Table S1). These isolates represented eight different lineages of the MTBC, including ancient (n: 11 L1, 2 L5, 7 L6), modern (n: 20 L2, 5 L3, 27 L4 including H37Rv and H37Ra) and one from each of *M. bovis* BCG and L8 (see Methods and Supplementary Table S1 for detailed information). The maximum SNP distance differences by lineage were > 350 SNPs, ensuring there was genetic diversity among isolates. All genomes were aligned to the reference H37Rv, and a total of 20,144 polyallelic sites and 6,632 indels were identified genome-wide across the 73 isolates. Differences in per SNP and indel nucleotide diversity (π) and absolute divergence (*dxy*) between the ancient and the modern lineages were observed in genomic regions containing *pe*/*ppe* genes (Figure 1A). The regions where high SNP or indel diversity was observed (π >9×10^−4^) coincided with highly homologous co-localized genes and recombination hotspots (*ppe3*, *pe_pgrs3*/*4*), or previously described highly diverse loci (*ppe1*, *pe_pgrs9*/*10*, *pe_pgrs50*, *pe_pgrs53*-*57*, *ppe55*, *ppe57-59*) (Phelan et al., 2016), where diversity was found suggestive of lineage-specific structural patterns.

Overall, a higher mean diversity across the whole-genome was obtained among ancient lineages (SNP *π* = 3.96×10^−4^; indel *π* = 9×10^−5^) than within modern lineages (SNP *π* = 2.31×10^−4^; indel *π* = 6×10^−5^), despite L4 having a high value of SNP π (Supplementary Figure S2). There was significantly higher SNP and indel diversity in *pe*/*ppe* genes compared to other gene functional groups (*p* < 0.01; Supplementary Table S2). Similarly, *dxy* for both SNPs and indels between the ancient and modern lineages was significantly higher in the *pe*/*ppe* gene families compared to other functional groups (*p* < 0.01; Supplementary Table S2), suggesting its genetic diversity contributes to lineage differentiation and can potentially classify MTBC lineages. Maximum-likelihood phylogenetic trees constructed using the genome-wide SNPs and indels resulted in the expected clustering by lineage (Figures 1B,C).

### 3.2. Conservation and disruption of the *pe* and *ppe* families across the MTBC lineages

**Figure 2:**
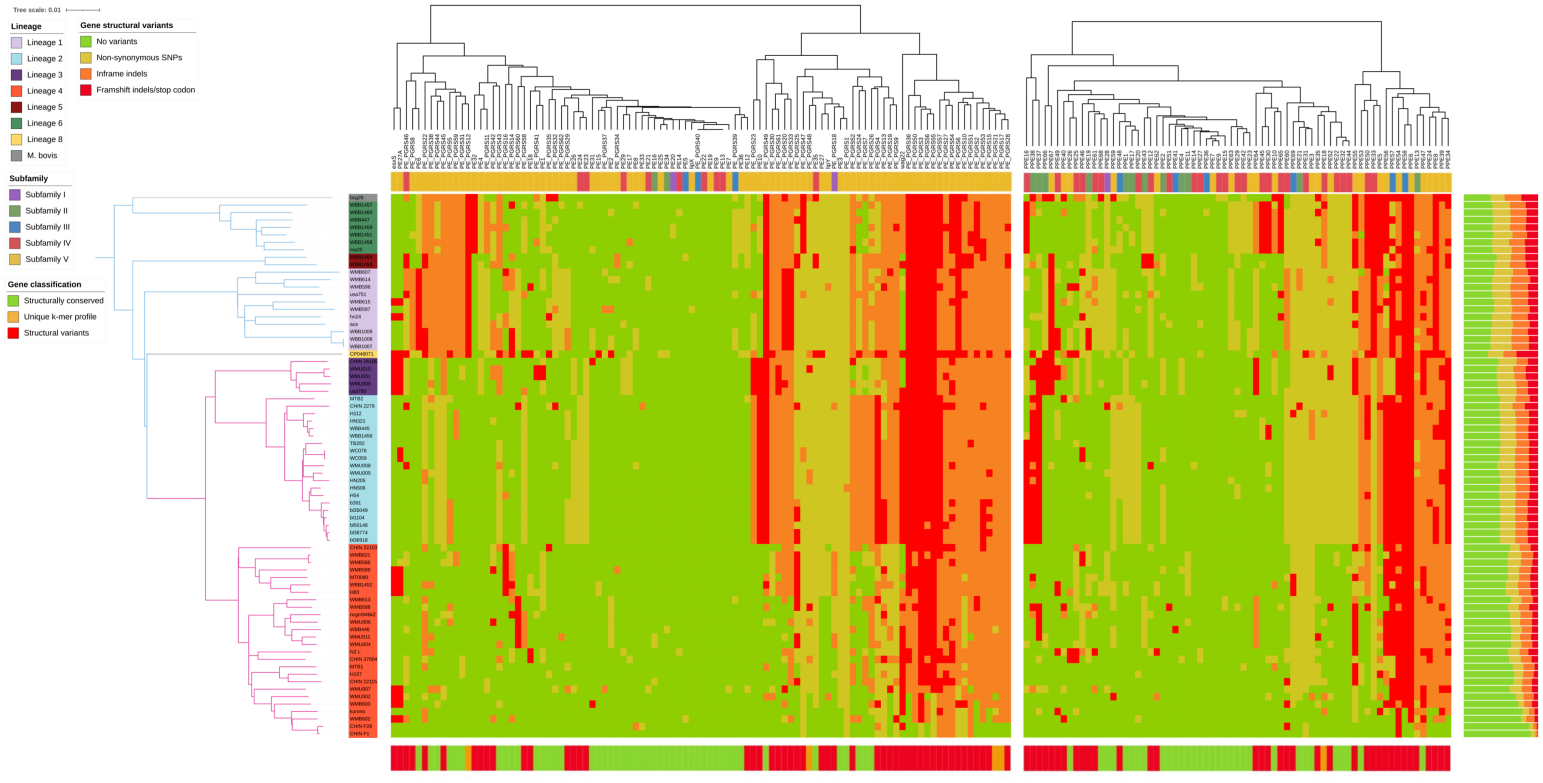
Heatmap showing the structural classification of each gene for each sample. Each row represents a separate sample, following the order based on the phylogenetic tree shown on the left. Genes on columns, pe family on the left, ppe family on the right. In green, genes without variants or synonymous SNPs; in yellow, genes with non-synonymous SNPs; in orange, genes with in-frame indels; in red, genes with frameshifts, changes in start/stop codons or large deletions. Top track shows the sub-family of each gene based on a previous classification (Gey van Pittius et al., 2006). Bottom track summarizes the structural classification of each gene across all samples in one of the following categories: structurally conserved (class C) in green, structural variants (class S) in red and unique k-mer profile (class K) in yellow. Barplot on the right shows the distribution of genes with each type of variant by sample.

Individual alignments for the *pe*/*ppe* genes were obtained (see Methods) to overcome the mapping problems in their repetitive and GC rich regions. The level of disruption caused by variants, relative to the H37Rv reference, was assigned for each isolate and each gene (see Methods and Supplementary Figure S1). The number of truncated or absent *pe*/*ppe* genes per lineage varied from 4 (L4.9) to ≥30 in the most distant lineages (L5/6/8 or *M. bovis* BCG), while the number of genes with complete conserved protein sequences per lineage was on average 109 for L4, decreasing to 60 for the most distant lineages on the phylogenetic tree (Figure 2). Overall, isolates had >55% of their *pe*/*ppe* genes relatively conserved, only harboring non-synonymous SNPs at most (median 118, range 93–163). Additionally, the 169 *pe*/*ppe* genes were classified into three different classes based on the presence of structural variants, namely those are: (i) conserved (C) (79/169; 27 *pe*, 20 *pe_pgrs* and 32 *ppe*), (ii) structurally non-conserved (S) (85/169; 9 *pe*, 40 *pe_pgrs* and 36 *ppe*), and (iii) with a unique *k-mer* profile (K) (5/169; 4 *pe_pgrs* and 1 *ppe*) (see Methods, Supplementary Tables S3, S4; Supplementary Figure S1). The genes in class K were those with large numbers of polymorphisms that could not be classified otherwise. Based on this classification, 46% of the *pe*/*ppe* genes were found conserved across the MTBC lineages analyzed (for a list of conserved genes see Supplementary Table S5).

To support the classification of the genes into the three classes, we analyzed short-read sequencing data from ~30 k dataset (Napier et al., 2020). Mean normalized coverage of the *pe*/*ppe* genes (0.74) was found to be lower than the rest of the genome (0.93; *p* < 0.01). There was the expected depletion in coverage in repetitive regions; however, not all *pe*/*ppe* genes fell in coverage blind spots. Because of their repetitive regions, both class K and S *pe*/*ppe* genes had lower mean coverage (combined: 0.67) compared to class C (0.82) or the rest of the genome (0.93; *p* < 0.01; Supplementary Figure S3A). Identifying the genes with lowest coverage values revealed 70 *pe*/*ppe* genes in troughs of low coverage corresponding to regions of high SNP and indel diversity observed earlier (Supplementary Figure S3B). The 20 genes with lowest coverage had been classified into the two non-conserved classes (S, K), highlighting difficulties in robustly characterizing their variants using a short-read alignment approach.

Subfamilies V (as defined by Gey van Pittius et. al. (Gey van Pittius et al., 2006)) of *pe*/*ppe* genes (mainly formed by *pe_pgrs* and *ppe_mptr*) are known to carry the most repetitive and polymorphic regions. Within the *pe* family, although 41% of the genes in subfamily V were structurally conserved, including 20 *pe_pgrs* (Supplementary Figure S4A), most of the structural diversity found across our isolates was observed in this group. Frequent differences in predicted protein lengths were found driven by deletions (Supplementary Figure S4B). Interestingly, the *dN*/*dS* ratio in *pe_pgrs* was 0.57 compared to 1.20 in the rest of the *pe* genes, suggesting negative selection effects. In contrast with the *pe* family, *ppe* subfamilies II and III harbored disruptive variants, including frameshifts, IS*6110* insertions, or other changes in the open reading frame (ORF) leading to gene fusions (Supplementary Figure S4C). Subfamily V *ppe_mptr* genes accounted for the highest level of disruption in protein sequence, with 16 genes in class S (16/24). In general, the largest variation in gene length was given by different numbers of the pentapeptide repeat (MPTR domain) and the integration of the IS*6110* insertion (Supplementary Figure 4D).

### 3.3. Nucleotide diversity in *pe*/*ppe* genes

**Figure 3:**
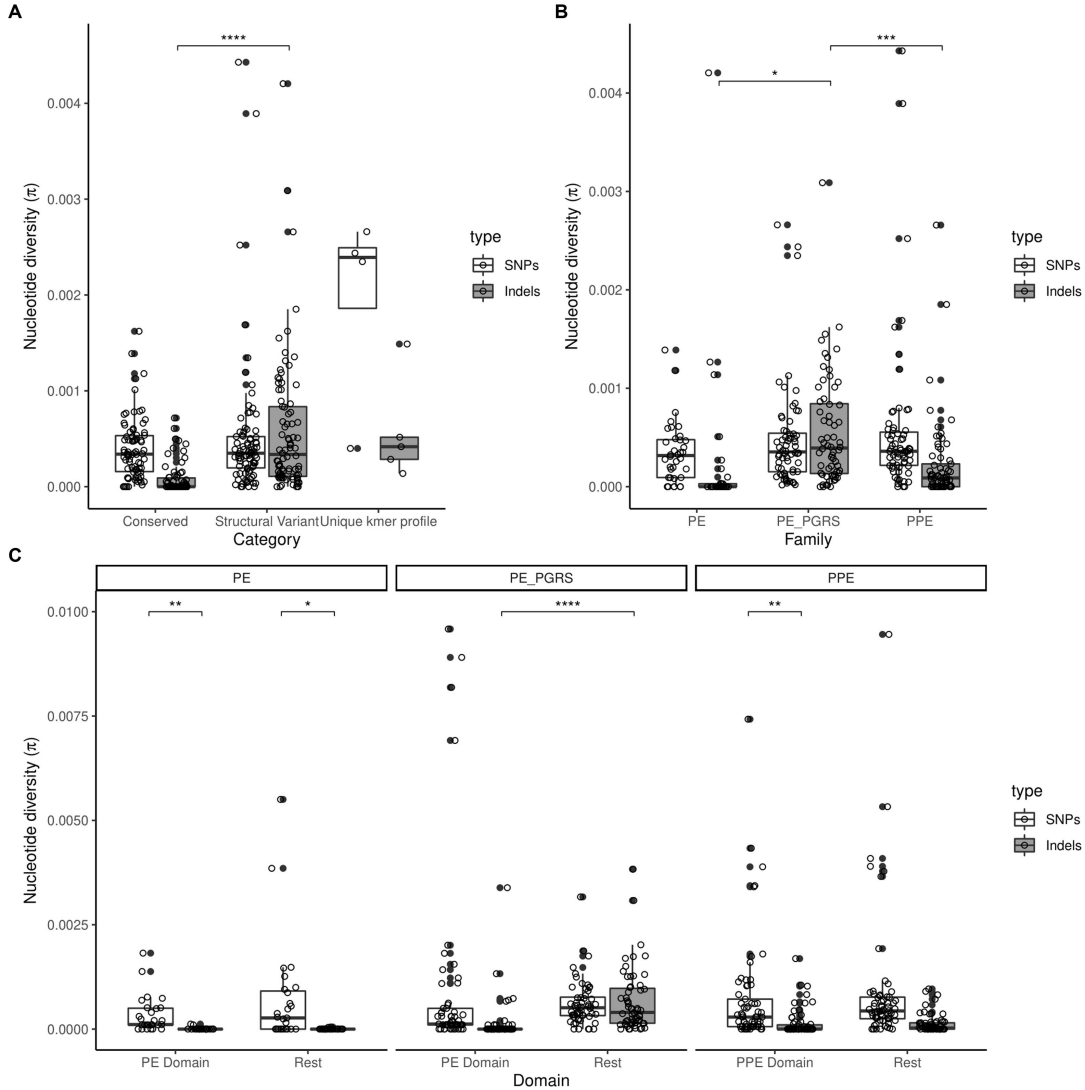
Boxplots of SNP and indel diversity in the 169 *pe/ppe* genes compared by **(A)** gene classification; **(B)** gene family and **(C)** domain within gene family. Outliers with *π* > 0.005 in **(A)** and **(B)** and *π* > 0.01 in **(C)** have been removed from figure. Adjusted *p*-value at (*) 5%, (**) 1%, (***) 0.1% or (****) 0.01% significance levels.

SNP and indel diversity were calculated for the 169 *pe*/*ppe* gene sequence alignments across the classes (C, S, K) (Supplementary Tables S3, S4). As expected, indel diversity in genes from class S was significantly higher than in class C (S mean indel π = 5.85×10^−4^, C mean indel *π* = 8.3×10^−5^; *p* < 0.001; Figure 3A). However, there were no significant differences of SNP π between classes. SNP π was heterogenous among class C and S genes (range 0 to >0.002). As expected, due to its polymorphic nature, the class K genes (*n* = 5) had a higher SNP diversity (mean SNP *π* < 7×10^−4^). A very weak correlation between SNP and indel diversity at a gene level was found (Spearman’s rho = 0.042; Supplementary Figure S5).

Overall, the *pe_pgrs* subfamily accounted for the majority of the indel diversity compared to other *pe* and *ppe* genes (Figure 3B), but diversity in the individual genes varies significantly (range indel π from <2×10^−5^ to >2×10^−3^). Interestingly, among *ppe* gene subfamilies, *ppe_svp* (IV) genes showed higher values of SNP and indel π than *ppe_mptr* (V) (Supplementary Figure S5). In accordance with the rest of the genome, *pe*/*ppe* genes in ancient lineages had a higher SNP π than modern counterparts (ancient mean SNP *π* = 6.7×10^−4^; modern mean SNP *π* = 4.2×10^−4^). Intra-lineage diversity was studied for those genes with representatives in greater than 5 lineages. There was a total of 34 and 32 genes where SNP or indel diversity, respectively, was zero for at least 4 of the 5 lineages studied, representing a situation where diversity is being driven by a single lineage or inter-lineage differences. Diversifying selection was found in 19 *pe*, 16 *pe_pgrs* and 19 *ppe* genes (*dN*/*dS* > 1.5; genome-wide average 0.71). Despite showing selection pressure, thirty of these genes belonged to class C, without structural variants (Supplementary Tables S3, S4). Genome-wide, only the “insertion sequences” functional (gene ontology) group showed a *dN*/*dS* ratio > 1 suggesting positive selection.

Diversity in the different domains showed that PE and PPE domains have low indel diversity (Figure 3C), suggesting specific structural conservation. In addition, these PE and PPE domains had a higher SNP nucleotide diversity than indel diversity (*p* < 0.01) except in the *pe_pgrs* subfamily. Indel diversity was greater after the PE domain in *pe_pgrs* genes (*p *< 0.01), while diversity in the *ppe* genes and the rest of the *pe* family was predominantly a result of SNPs.

### 3.4. Large insertions

Class S genes had a high abundance of large insertions that could be distinguished into two types: (i) >25 bp insertions with >70% identity to same or other *pe*/*ppe* genes, and (ii) the integration of the IS*6110* insertion sequence. The former corresponds mostly to sequences of repetitive regions in *pe_pgrs* and *ppe_mptr* genes, which could result from homologous recombination, and follow a lineage-specific pattern in several cases. Duplications were also identified, including an extra copy of *ppe53* found in all isolates except L4.3 to L4.9 and L8 with the same N-terminal but different C-terminal domain (Supplementary Figure S6). The integration of IS*6110* was observed in regions around *pe*/*ppe* genes, which were similar across the different lineages (Supplementary Figure S7). Thirteen genes (1 *pe* and 12 *ppe*, including 9 *ppe_mptr*) were found to harbor IS*6110* in at least one isolate (Supplementary Table S6), which was responsible for the disruption of the protein sequence. Genes known to have IS*6110* inserted, such as *ppe34*, had a high frequency of integration (34 isolates, including all L2-3), leading to two shorter ORFs compared to H37Rv-PPE34 (Supplementary Figure S8), confirmed with PGAP annotation. The *ppe38-40* loci are a hotspot for the insertion of IS*6110*. This genomic region as annotated in the H37Rv reference is rarely found in clinical isolates, but one often encounters the *ppe71* duplication (Ates et al., 2018). We observed the two *esx* flanking genes and *ppe71* in many isolates (*n* = 38/72; 52.8%), including the laboratory strains H37Rv and H37Ra (Supplementary Figure S9). Nevertheless, all Beijing (L2.1.1) isolates had only a single copy, which was truncated losing the PPE domain by the insertion of IS*6110*, and has been found to suppress the secretion of PE_PGRS and PPE_MPTR proteins (Ates et al., 2018). The contiguous gene, *ppe39*, has been described in an extended version in Beijing isolates (Han et al., 2015). Most isolates (except H37Rv/Ra and L4.6/L4.9) had an extra ~268 residues at the N-terminal, which included a PPE domain that appears truncated by the integration of IS*6110* in the reference genome.

### 3.5. Other gene fusions and duplications

**Figure 4:**
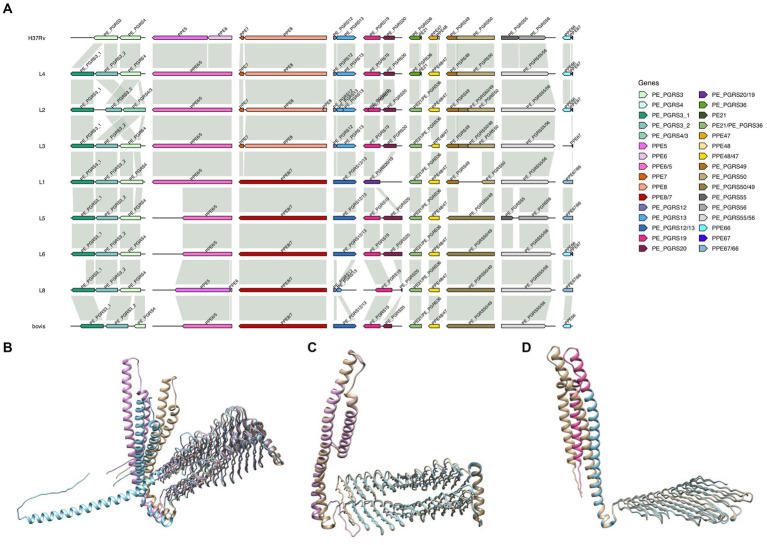
**(A)** Gene organization of 10 pairs of consecutive genes where variants modify the open reading frame generating a gene fusion in at least one lineage. Gene organization shown with representatives for each lineage; * only in some isolates from the lineage. **(B)**, **(C)** and **(D)** Predicted protein structures by AlphaFold of **(B)** PE_PGRS4/3, **(C)** PE_PGRS12/13 and **(D)** PE21/PE_PGRS36. In beige, structure of the fused protein; in blue PE_PGRS4 (B), PE_PGRS13 **(C)** and PE_PGRS36 (D); in pink PE_PGRS3 (B), PE_PGRS12 (C) and PE21 (D).

We found 10 pairs of *pe*/*ppe* genes that showed potential gene fusions compared to the H37Rv reference, including the fusion of the PE and PGRS domains of adjacent genes. The *pe_pgrs4*/*3* (L2) and *pe_pgrs20*/*19* (L1) loci are two examples of the fusion of domains in single lineages, where a large deletion covering the end of the upstream gene leads to the merging of these two adjacent genes forming a *pe_pgrs* gene (Figure 4A). Using AlphaFold software, the predicted protein structure of the PE_PGRS4/3 fusion in L2 revealed a PE_PGRS protein highly similar to PE_PGRS3 and PE_PGRS4 (Figure 4B). For *ppe6*/*5*, *ppe8*/*7*, *pe_pgrs12*/*13*, *pe_pgrs50*/*49*, *pe_pgrs55*/*56* and *ppe67*/*66* gene pairs, the ORF continued until the end of the second gene due to a frameshift caused by a small indel (Figure 4A). Interestingly, *pe_pgrs12* and *pe_pgrs55* have a PE domain, while in the downstream genes *pe_pgrs13* and *pe_pgrs56* this domain is absent, only showing PGRS motifs, and therefore, their combination leads to a PE_PGRS-like structure inferred by AlphaFold software (Figure 4C). Likewise, for *ppe6*/*5* and *ppe8*/*7*, the *ppe5* and *ppe7* loci do not have any PPE domain, thereby the gene fusion leads to a PPE_MPTR-like structure. Finally, there are four *pe*/*ppe* genes in *Mtb* annotated as pseudogenes (*pe21*/*pe_pgrs36* and *ppe48*/*ppe47*), where small indels causing frameshifts led to a change in ORF and the consequent formation of single PE_PGRS-or PPE_MPTR-like genes (Figure 4A). All these gene fusions were confirmed by PGAP annotation.

The *pe_pgrs3* locus is a recombination hotspot (Phelan et al., 2016), and several large indels were identified when aligned to the H37Rv reference, including insertions linked to duplication of repetitive regions. These observations confirm the non-conserved nature of the *pe_pgrs3* gene, and make it difficult to characterize with the usual variant calling pipelines. Surprisingly, the protein sequences obtained from the aligned region showed a duplication of *pe_pgrs3* in almost every sample analyzed (Figure 4A), confirmed by the annotation of the assemblies obtained by PGAP. The two *pe_pgrs3* genes identified were highly similar differing in the presence/absence of the C-terminal domain from H37Rv-*pe_pgrs3* gene, which also shows lineage-specific patterns. Despite the lack of concordance with the reference, we observed a significant degree of conservation within lineages. The *pe_pgrs3* gene is duplicated in *M. bovis* and *M. canetti*, and until now, it was believed not to be duplicated in *Mtb* (De Maio et al., 2020). Laboratory strains (H37Rv and H37Ra) retained a unique gene, which combines N-terminal and C-terminal from the ancestral two copies, while the other lineages carried two copies of the gene differing from *M. bovis* (L1, L3 or L4) in the C-terminal domain of one of the copies (Supplementary Figure S10).

### 3.6. Lineage-specific SNPs and indels in *pe*/*ppe* genes

A total of 3,649 SNPs and 1,319 indels were identified among the *pe*/*ppe* genes, from which 459 SNPs and 122 indels were found in the class C genes. A completely conserved protein sequence across all lineages was only found for seven genes (*ppe7*, *pe9*, *pe13*, *pe19*, *pe22*, *pe25* and *pe_pgrs40*), including *ppe7* that differed from the H37Rv reference by a 1 bp insertion. The existence of inter-without intra-lineage diversity in some genes suggested a potential lineage-specific pattern. We performed a principal component analysis with SNPs and indel matrices for each sample (excluding L8). Clustering by lineage was clear for indels, and with sub-groups for some lineages being observed using SNPs (Supplementary Figure S11). Following the hypothesis of having lineage-specific markers within these genes, we built three maximum likelihood phylogenetic trees with only these SNPs, indels, and both (Supplementary Figure S12). Sixteen genes with >1.5% of its coding region being polymorphic sites or with a unique *k-mer* profiler were removed to reconstruct the SNP tree. The topologies of the trees obtained with SNPs and indels were different; however, both showed a clear clustering by lineage, suggesting lineage-specific patterns.

The fixation index (*F_ST_*) was calculated to identify these lineage-specific polymorphisms, comparing one lineage against the others for each of the variants found in *pe*/*ppe* genes across the 72 available genomes. Overall, 83 SNPs and 8 indels (including SNPs and frameshifts leading to disrupted proteins) were identified with an *F_ST_* of 1 in one lineage within our dataset and with an allele frequency > 0.95 in the corresponding lineage within the ~30 k dataset (Table 1; Supplementary Table S7).

**Table 1:** Lineage-or clade-specific variants.

Lineage	Class C Gene [mutation]	Class S Gene [mutation]	Class K Gene [mutation]
Ancient	*ppe4* [A185A], *ppe28* [C144W], *pe_pgrs44* [G478G]	*ppe5* [S1765F], *ppe8* [I3250F; 9889_9890insATA***], *ppe12* [R545K], *pe_pgrs14* [A246A], *pe_pgrs47* [G383G]	
L1	*pe4* [K164N], *ppe2* [T412T], *pe_pgrs5* [G225D], *pe_pgrs7* [G951R], *pe_pgrs11* [G280R], *ppe13* [G336G], *ppe44* [G59V], *ppe61* [T257M], *ppe63* [Y365N]	*ppe5* [I1273V], *ppe8* [139_139del, V118A], *pe_pgrs14* [G668D], *pe_pgrs47* [S20S], *ppe64* [G306S]	
L2	*ppe17* [P167L], *pe14* [A106A], *pe16* [A96A], *pe24* [G216V], *pe_pgrs58* [A314V]	*pe_pgrs22* [G730G]	
L3	*pe3* [S175P], *pe4* [F197S], *ppe4* [L52M], *ppe10* [W147S], *pe_pgrs7* [G405G], *pe17* [T285I], *pe_pgrs30* [T600N], *pe26* [S330L], *ppe48* [I64L]	*pe1* [G369R], *ppe5* [G960A], *ppe8* [D741N, S1920F], *pe_pgrs6* [A124V], *pe_pgrs10* [G799G], *ppe19* [F4V], *ppe33* [G22S], *ppe54* [G2189S], *ppe64* [63_64del*]	*pe_pgrs45* [G437G]
L2/3		*pe10* [337_337del****]	
L4		*lipY* [A58G]	
L4.1		*pe_pgrs16* [1968_1969insG*]	
L5	*ppe2* [E140G, D431N], *ppe3* [E448D], *ppe14* [T293M], *pe12* [L217F], *pe_pgrs24* [L101R], *pe_pgrs30* [R115L], *ppe29* [A366P], *ppe31* [H188Y], *ppe36* [I25I], *pe_pgrs39* [A109T], *pe_pgrs40* [D29D], *pe26* [G160S], *pe_pgrs44* [A439A], *pe_pgrs59* [G22D]	*ppe8* [F414V], *ppe12* [G378S], *pe_pgrs22* [G118G], *ppe16* [G349R], *pe_pgrs23* [G280G], *ppe24* [S716R], *pe_pgrs32* [E76D; A483T], *ppe37* [V124M], *pe_pgrs42* [G125G], *ppe43* [449_454del*], *lipY* [F129S], *pe_pgrs55* [1411_1411del*]	*ppe18* [H234R]
L6	*ppe1* [P298P], *ppe3* [M450T]*, ppe10* [G288A], *ppe13* [N244N], *ppe23* [S37P], *pe_pgrs43* [W1503R]	*pe1* [P494L], *ppe16* [1279_1283del*], *ppe45* [W75*^(^*^)^], *ppe56* [6586_6586del*]	
*M. bovis*	*pe3* [P255T], *ppe10* [W8*^(^*^)^], *ppe20* [V94A], *pe_pgrs30* [A172V]	*pe1* [G26R], *ppe8* [G2403G], *pe_pgrs15* [L113L], *ppe25* [925_927del**], *pe_pgrs41* [S26N]	

*Truncated protein; **In-frame; ***Leads to gene fusion; ****Delayed stop codon.

Differences between H37Rv annotation and the rest of genomes were also found in the PGAP annotation calls. The differences were mostly due to the lineage diversity, duplications or gene fusions already discussed; however, some small sequences (≤500 bp) were identified in some samples as *pe/ppe* family proteins. In brief, three small sequences were found upstream of *ppe59* in various samples of different lineages, and all L3 and almost all L2 had a sequence (492–647 bp) predicted as PE family protein upstream of *pe_pgrs35*. In L8, a 1,665 bp sequence not present in any other lineage was predicted as PE family protein, which corresponds with one of the “new *pe*/*ppe* genes” discovered in L8 (Ngabonziza et al., 2020).

## 4. Discussion

The *pe* and *ppe* genes are important MTBC loci, but are routinely excluded form whole-genome sequencing studies, especially those using short sequence data, due to difficulties in accurately mapping their repetitive and polymorphic regions (Meehan et al., 2019). In a recent study, Marin et al. (2022) found several *pe*/*ppe* genes with good mappability and variant detection with short-read sequencing platforms that could be included in WGS analysis with confidence. The majority of these genes belonged to class C in our analysis, congruent with a better coverage of the conserved *pe*/*ppe* genes found also in previous studies (Gómez-González et al., 2022), while class K genes were found with lower scores for Illumina mappability. Long read sequencing technologies can be of use to overcome this problem (Gómez-González et al., 2022). We analyzed PacBio assemblies to provide the most comprehensive picture to date of genetic diversity in all 169 *pe*/*ppe* genes. The sequence analysis revealed a large amount of both conservation and diversity across the *pe*/*ppe* families. As expected, we observed greater nucleotide diversity in *pe*/*ppe* genes compared to the rest of the genome, especially in some clustered loci (e.g., *pe_pgrs53-57*, *ppe57-59*), with some of them predicted to be pathogenicity islands (Xie et al., 2014). The diversity is driven not only by SNPs but also by indels, including the integration of IS*6110*, for which several transposition sites have been identified among *pe/ppe* genes, especially within members of the *ppe* subfamily V (*ppe_mptr*) (Yesilkaya et al., 2005; Namouchi and Mardassi, 2006; McEvoy et al., 2009, 2012; Reyes et al., 2012). Consistent with previous findings (Reyes et al., 2012), we observed a tendency of occurrence of IS*6110* insertions in genomic regions with *pe*/*ppe* genes, including some genes exhibiting lineage-specific patterns. The *ppe38-40* represents a known hotspot for IS*6110* integration with consequences for strain virulence (McEvoy et al., 2009; Ates et al., 2018). SNP and indel diversity were heterogeneous across the genes. The class S genes displayed greater indel diversity but a similar SNP diversity to class C. The main source of diversity in *pe_pgrs* genes was identified after the PE domain, mainly driven by indels. In contrast, diversity was more often the result of SNPs in other *pe*/*ppe* genes. In line with previous work, the *dN*/*dS* ratios obtained broadly varied across individual genes across *pe/ppe* families (Copin et al., 2014).

Evidence of homologous recombination, especially in repetitive regions of *pe*/*ppe* (Karboul et al., 2008; Phelan et al., 2016), and events of gene conversion (Karboul et al., 2006) have been described. For example, homologous recombination due to repetitive nature of the PGRS domain has been previously suggested to occur in the *pe_pgrs4*/*3* locus (Phelan et al., 2016), but no duplications of these genes have been previously described. We found a second copy of *pe_pgrs3* in most of the isolates, with a similar configuration of the one found in *M. bovis* and *M. canetti* (De Maio et al., 2020). Due to the similarity to the ancestral configuration, it is possible that recombination events have resulted in the loss of one copy in H37Rv and related strains, which could be suggestive of reductive evolution in *Mtb.* Several gene fusions compared to the annotated H37Rv were also identified in this study. Some genes were found in single lineages (e.g., *pe_pgrs20*/*19*), while others were in all isolates (e.g., *ppe48*/*47*). Interestingly, the four *pe*/*ppe* genes annotated as pseudogenes, organized in two operons in H37Rv, were found to form a single ORF in most isolates leading to a potentially functional protein. This lack of consistency between the H37Rv annotated sequences and the predicted protein sequences in the clinical isolates could potentially mislead and hinder the capture of variants when using sequence alignment methods. Given the lineage-specific structural variants observed, the use of lineage reference genomes could be a possibility to overcome the difficulties in capturing variants in genes where variation must be being missed due to substantial differences with the H37Rv reference, potentially improving the mappability with short-read sequencing methods. Other studies (Chitale et al., 2022) have also suggested the use of revised reference genomes of H37Rv including changes in some *pe*/*ppe* genes found, which would overall improve the accuracy of variant detection.

The inter-lineage diversity found in some *pe*/*ppe* genes, together with its substantial impact on the phylogenetic differences between the ancient and the modern lineages, suggested the presence of lineage-specific variants in these regions that could be phylogenetically informative. We identified numerous lineage-specific SNPs and indels validated in ~ 30 k *Mtb* isolates with whole-genome sequencing data. Protein disruption was a frequent outcome of the lineage-specific indels, which considering the putative role in host-pathogen interactions of these proteins, could provide insights into different behavior between strains. One limitation of short-read sequencing data for the validation work was the lack of accuracy in detecting big indels, especially among repetitive regions.

All *pe*/*ppe* genes were classified based on the conservation observed across the 72 isolates. Structural variants, such as frameshifts, changes in start and stop codons, and large deletions, were responsible for the classification of numerous genes as non-conserved, which often were identified across one or multiple sub-lineages, showing an otherwise conserved profile within lineage. Member of subfamilies V were found in higher numbers among the non-conserved genes. Importantly, this classification was based on the alignment to the H37Rv sequence, which, as shown, does not always represent the functional locus. However, on average, more than half of the *pe*/*ppe* gene members per sample were found to be conserved, suggesting an important role. The various levels of diversity that different genes display have been proposed to imply non-redundant functions (Copin et al., 2014). Nevertheless, the complex gene layout found in the different lineages requires more investigation to understand the functional consequences of the variation observed. One difficulty is the lack of structural data for *pe/ppe* proteins that restricts the prediction of functional consequences. However, novel *in silico* tools, such as AlphaFold software (Jumper et al., 2021), can be of assistance.

Some *pe/ppe* proteins have demonstrated to act as modulators of the immune response and consequently, multiple epitopes have been characterized on these proteins being investigated as targets for vaccine development (Medha and Sharma, 2021). T-cell epitopes are found in the conserved PE domains of *pe_pgrs* genes rather than the variable sequences, supporting the hypothesis that this conservation favors infection (Copin et al., 2014; Fishbein et al., 2015). It is plausible that gene fusions where loci with missing *pe/ppe* domains are transcribed with an upstream locus lead to a functional protein. On this premise, understanding the structural diversity of these genes and its consequent effect on these proteins is crucial for its potential use in vaccine development, which ideally would target conserved sequences across the different lineages. Homolka et al. (2016) showed how diversity in *ppe18* could significantly impact the effectivity of a vaccine candidate. Additionally, the role of these proteins in cell wall localisation and small molecule transportation means they could be explored as drug targets (Ates, 2020).

In conclusion, although there is significant variation in *pe/ppe* genes, with this study we have found that some are relatively conserved. We have provided a list of conserved genes that could be included in whole-genome sequencing analysis rather than being excluded, especially as they can be phylogenetically informative. These proteins play an essential role in host-pathogen interactions, and therefore it is important to elucidate their function and the potential impact of diversity on pathogenicity and virulence. Future studies in a larger number of isolates, combined with functional characterization, will lead to insights that can assist with the control of the tuberculosis disease.

## Data availability statement

The datasets presented in this study can be found in online repositories. The names of the repository/repositories and accession number(s) can be found in the article/Supplementary material.

## Author contributions

SC, JP, and TGC conceived and directed the project. PG-G and AG undertook sample processing and DNA extraction. PG-G performed bioinformatic and statistical analyzes under the supervision of SC, JP, and TGC. LT provided data. SC led the generation of sequence data, with assistance from MH and LT. PG-G, SC, JP, and TGC interpreted results. PG-G wrote the first draft of the manuscript with inputs from JP and TGC. PG-G, JP, and TGC compiled the final manuscript. All authors contributed to the article and approved the submitted version.

## References

[ref1] Abdallah A. M. Gey van Pittius N. C. DiGiuseppe Champion P. A. Cox J. Luirink J. Vandenbroucke-Grauls C. M. J.. (2007). Type VII secretion–mycobacteria show the way. Nat. Rev. Microbiol. 5, 883–891. doi: 10.1038/nrmicro1773, PMID: 17922044

[ref2] Akhter Y. Ehebauer M. T. Mukhopadhyay S. Hasnain S. E. (2012). The *pe/ppe* multigene family codes for virulence factors and is a possible source of mycobacterial antigenic variation: perhaps more? Biochimie 94, 110–116. doi: 10.1016/j.biochi.2011.09.026, PMID: 22005451

[ref3] Ates L. S. (2020). New insights into the mycobacterial PE and PPE proteins provide a framework for future research. Mol. Microbiol. 113, 4–21. doi: 10.1111/mmi.14409, PMID: 31661176 PMC7028111

[ref4] Ates L. S. Dippenaar A. Ummels R. Piersma S. R. Van der Woude A. D. Van der Kuij K.. (2018). Mutations in ppe38 block PE_PGRS secretion and increase virulence of *Mycobacterium tuberculosis*. Nat. Microbiol. 3, 181–188. doi: 10.1038/s41564-017-0090-6, PMID: 29335553

[ref5] Charif D. Lobry J. R.. (2007). *Seqin R 1.0–2: A contributed package to the R project for statistical computing devoted to biological sequences retrieval and analysis. In: Structural approaches to sequence evolution biological and medical physics, biomedical engineering*. pp. 207–232.

[ref6] Chitale P. Lemenze A. D. Fogarty E. C. Shah A. Grady C. Odom-Mabey A. R.. (2022). A comprehensive update to the *Mycobacterium tuberculosis* H37Rv reference genome. Nat. Commun. 13, 1–12. doi: 10.1038/s41467-022-34853-x 36400796 PMC9673877

[ref7] Cole S. T. Brosch R. Parkhill J. Garnier T. Churcher C. Harris D.. (1998). Deciphering the biology of *Mycobacterium tuberculosis* from the complete genome sequence. Nature 393, 537–544. doi: 10.1038/31159, PMID: 9634230

[ref8] Copin R. Coscollá M. Seiffert S. N. Bothamley G. Sutherland J. Mbayo G.. (2014). Sequence diversity in the pe_pgrs genes of *Mycobacterium tuberculosis* is independent of human T cell recognition. MBio 5:e00960. doi: 10.1128/mBio.00960-13, PMID: 24425732 PMC3903279

[ref9] Coscolla M. Gagneux S. (2014). Consequences of genomic diversity in *Mycobacterium tuberculosis*. Semin. Immunol. 26, 431–444. doi: 10.1016/j.smim.2014.09.012, PMID: 25453224 PMC4314449

[ref10] De Maio F. Berisio R. Manganelli R. Delogu G. (2020). PE_PGRS proteins of *Mycobacterium tuberculosis*: a specialized molecular task force at the forefront of host–pathogen interaction. Virulence 11, 898–915. doi: 10.1080/21505594.2020.1785815, PMID: 32713249 PMC7550000

[ref11] Elghraoui A. Modlin S. J. Valafar F. (2017). SMRT genome assembly corrects reference errors, resolving the genetic basis of virulence in *Mycobacterium tuberculosis*. BMC Genomics 18:302. doi: 10.1186/s12864-017-3687-5, PMID: 28415976 PMC5393005

[ref12] Fishbein S. van Wyk N. Warren R. M. Sampson S. L. (2015). Phylogeny to function: pe/ppe protein evolution and impact on *Mycobacterium tuberculosis* pathogenicity. Mol. Microbiol. 96, 901–916. doi: 10.1111/mmi.12981, PMID: 25727695

[ref13] Gey van Pittius N. C. Sampson S. L. Lee H. Kim Y. Van Helden P. D. Warren R. M. (2006). Evolution and expansion of the *Mycobacterium tuberculosis* PE and PPE multigene families and their association with the duplication of the ESAT-6 (esx) gene cluster regions. BMC Evol. Biol. 6:95. doi: 10.1186/1471-2148-6-95, PMID: 17105670 PMC1660551

[ref14] Gómez-González P. J. Campino S. Phelan J. E. Clark T. G. (2022). Portable sequencing of *Mycobacterium tuberculosis* for clinical and epidemiological applications. Brief. Bioinform. 23, 1–10. doi: 10.1093/bib/bbac256 PMC9487601 35894606

[ref15] Gomez-Gonzalez P. J. Andreu N. Phelan J. E. De Sessions P. F. Glynn J. R. Crampin A. C.. (2019). An integrated whole genome analysis of *Mycobacterium tuberculosis* reveals insights into relationship between its genome, transcriptome and methylome. Sci. Rep. 9, 1–11. doi: 10.1038/s41598-019-41692-2, PMID: 30914757 PMC6435705

[ref16] Han S. J. Song T. Cho Y. J. Kim J. S. Choi S. Y. Bang H. E.. (2015). Complete genome sequence of *Mycobacterium tuberculosis* K from a Korean high school outbreak, belonging to the Beijing family. Stand. Genomic Sci. 10, 1–8. doi: 10.1186/s40793-015-0071-4 26473025 PMC4606834

[ref17] Homolka S. Ubben T. Niemann S. (2016). High sequence variability of the PPE18 gene of clinical *Mycobacterium tuberculosis* complex strains potentially impacts effectivity of vaccine candidate M72/AS01E. PLoS One 11, 1–10. doi: 10.1371/journal.pone.0152200 PMC4806982 27011018

[ref18] Jumper J. Evans R. Pritzel A. Green T. Figurnov M. Ronneberger O.. (2021). Highly accurate protein structure prediction with alpha fold. Nature 596, 583–589. doi: 10.1038/s41586-021-03819-2, PMID: 34265844 PMC8371605

[ref19] Karboul A. Mazza A. Gey van Pittius N. C. Ho J. L. Brousseau R. Mardassi H. (2008). Frequent homologous recombination events in *Mycobacterium tuberculosis* pe/ppe multigene families: potential role in antigenic variability. J. Bacteriol. 190, 7838–7846. doi: 10.1128/JB.00827-08, PMID: 18820012 PMC2583619

[ref20] Karboul A. Van Pittius N. C. G. Namouchi A. Vincent V. Sola C. Rastogi N.. (2006). Insights into the evolutionary history of tubercle bacilli as disclosed by genetic rearrangements within a PE_PGRS duplicated gene pair. BMC Evol. Biol. 6, 1–18. doi: 10.1186/1471-2148-6-107 17163995 PMC1762029

[ref21] Katoh K. Standley D. M. (2013). MAFFT multiple sequence alignment software version 7: improvements in performance and usability. Mol. Biol. Evol. 30, 772–780. doi: 10.1093/molbev/mst010, PMID: 23329690 PMC3603318

[ref22] Kolmogorov M. Yuan J. Lin Y. Pevzner P. A. (2019). Assembly of long, error-prone reads using repeat graphs. Nat. Biotechnol. 37, 540–546. doi: 10.1038/s41587-019-0072-8, PMID: 30936562

[ref23] Li H. Durbin R. (2009). Fast and accurate short read alignment with burrows-wheeler transform. Bioinformatics 25, 1754–1760. doi: 10.1093/bioinformatics/btp324, PMID: 19451168 PMC2705234

[ref24] Marin M. Vargas R. Harris M. Jeffrey B. Epperson L. E. Durbin D.. (2022). Benchmarking the empirical accuracy of short-read sequencing across the *M. tuberculosis* genome. Bioinformatics 38, 1781–1787. doi: 10.1093/bioinformatics/btac023, PMID: 35020793 PMC8963317

[ref25] McEvoy C. R. E. Cloete R. Müller B. Schürch A. C. Van Helden P. D. Gagneux S.. (2012). Comparative analysis of *Mycobacterium tuberculosis* pe and ppe genes reveals high sequence variation and an apparent absence of selective constraints. PLoS One 7:e30593. doi: 10.1371/journal.pone.0030593, PMID: 22496726 PMC3319526

[ref26] McEvoy C. R. Van Helden P. D. Warren R. M. Van Pittius N. C. G. (2009). Evidence for a rapid rate of molecular evolution at the hypervariable and immunogenic *Mycobacterium tuberculosis* PPE38 gene region. BMC Evol. Biol. 9, 237–221. doi: 10.1186/1471-2148-9-237 19769792 PMC2758852

[ref27] McGuire A. Weiner B. Park S. Wapinski I. Raman S. Dolganov G.. (2012). Comparative analysis of Mycobacterium and related actinomycetes yields insight into the evolution of *Mycobacterium tuberculosis* pathogenesis. BMC Genomics 13:120. doi: 10.1186/1471-2164-13-120, PMID: 22452820 PMC3388012

[ref28] Medha S. S. Sharma M. (2021). Proline-glutamate/proline-proline-glutamate (pe/ppe) proteins of *Mycobacterium tuberculosis*: the multifaceted immune-modulators. Acta Trop. 222:106035. doi: 10.1016/j.actatropica.2021.106035, PMID: 34224720

[ref29] Meehan C. J. Goig G. A. Kohl T. A. Verboven L. Dippenaar A. Ezewudo M.. (2019). Whole genome sequencing of *Mycobacterium tuberculosis*: current standards and open issues. Nat. Rev. Microbiol. 17, 533–545. doi: 10.1038/s41579-019-0214-5, PMID: 31209399

[ref30] Mistry J. Chuguransky S. Williams L. Qureshi M. Salazar G. A. Sonnhammer E. L. L.. (2021). Pfam: the protein families database in 2021. Nucleic Acids Res. 49, D412–D419. doi: 10.1093/nar/gkaa913, PMID: 33125078 PMC7779014

[ref31] Modlin S. J. Robinhold C. Morrissey C. Mitchell S. N. Ramirez-Busby S. M. Shmaya T.. (2021). Exact mapping of Illumina blind spots in the *Mycobacterium tuberculosis* genome reveals platform-wide and workflow-specific biases. Microb Genomics. 7:mgen000465. doi: 10.1099/mgen.0.000465, PMID: 33502304 PMC8190613

[ref32] Murrell B. Weaver S. Smith M. D. Wertheim J. O. Murrell S. Aylward A.. (2015). Gene-wide identification of episodic selection. Mol. Biol. Evol. 32, 1365–1371. doi: 10.1093/molbev/msv035, PMID: 25701167 PMC4408417

[ref33] Namouchi A. Mardassi H. (2006). A genomic library-based amplification approach (GL-PCR) for the mapping of multiple IS6110 insertion sites and strain differentiation of *Mycobacterium tuberculosis*. J. Microbiol. Methods 67, 202–211. doi: 10.1016/j.mimet.2006.03.021, PMID: 16725220

[ref34] Napier G. Campino S. Merid Y. Abebe M. Woldeamanuel Y. Aseffa A.. (2020). Robust barcoding and identification of *Mycobacterium tuberculosis* lineages for epidemiological and clinical studies. Genome Med. 12:114. doi: 10.1186/s13073-020-00817-3, PMID: 33317631 PMC7734807

[ref35] Ngabonziza J. C. S. Loiseau C. Marceau M. Jouet A. Menardo F. Tzfadia O.. (2020). A sister lineage of the *Mycobacterium tuberculosis* complex discovered in the African Great Lakes region. Nat. Commun. 11:2917. doi: 10.1038/s41467-020-16626-6, PMID: 32518235 PMC7283319

[ref36] Nguyen L. T. Schmidt H. A. Von Haeseler A. Minh B. Q. (2015). IQ-TREE: a fast and effective stochastic algorithm for estimating maximum-likelihood phylogenies. Mol. Biol. Evol. 32, 268–274. doi: 10.1093/molbev/msu300, PMID: 25371430 PMC4271533

[ref37] Pfeifer B. Wittelsbürger U. Ramos-Onsins S. E. Lercher M. J. (2014). Pop genome: an efficient swiss army knife for population genomic analyses in R. Mol. Biol. Evol. 31, 1929–1936. doi: 10.1093/molbev/msu136, PMID: 24739305 PMC4069620

[ref38] Phelan J. E. Coll F. Bergval I. Anthony R. M. Warren R. Sampson S. L.. (2016). Recombination in pe/ppe genes contributes to genetic variation in *Mycobacterium tuberculosis* lineages. BMC Genomics 17:151. doi: 10.1186/s12864-016-2467-y, PMID: 26923687 PMC4770551

[ref39] Phelan J. De Sessions P. F. Tientcheu L. Perdigao J. Machado D. Hasan R.. (2018). Methylation in *Mycobacterium tuberculosis* is lineage specific with associated mutations present globally. Sci. Rep. 8, 1–7. doi: 10.1038/s41598-017-18188-y, PMID: 29317751 PMC5760664

[ref40] Phelan J. E. O’Sullivan D. M. Machado D. Ramos J. Oppong Y. E. A. Campino S.. (2019). Integrating informatics tools and portable sequencing technology for rapid detection of resistance to anti-tuberculous drugs. Genome Med. 11:41. doi: 10.1186/s13073-019-0650-x, PMID: 31234910 PMC6591855

[ref41] Qian J. Chen R. Wang H. Zhang X. (2020). Role of the pe/ppe family in host–pathogen interactions and prospects for anti-tuberculosis vaccine and diagnostic tool design. Front. Cell. Infect. Microbiol. 10, 1–8. doi: 10.3389/fcimb.2020.594288, PMID: 33324577 PMC7726347

[ref42] Quinlan A. R. Hall I. M. (2010). BEDTools: a flexible suite of utilities for comparing genomic features. Bioinformatics 26, 841–842. doi: 10.1093/bioinformatics/btq033, PMID: 20110278 PMC2832824

[ref43] Reyes A. Sandoval A. Cubillos-Ruiz A. Varley K. E. Hernández-Neuta I. Samper S.. (2012). IS-seq: a novel high throughput survey of in vivo IS6110 transposition in multiple *Mycobacterium tuberculosis* genomes. BMC Genomics 13:249. doi: 10.1186/1471-2164-13-249, PMID: 22703188 PMC3443423

[ref44] Rizk G. Lavenier D. Chikhi R. (2013). DSK: K-mer counting with very low memory usage. Bioinformatics 29, 652–653. doi: 10.1093/bioinformatics/btt020, PMID: 23325618

[ref45] Sable S. B. Posey J. E. Scriba T. J. (2019). Tuberculosis vaccine development: Progress in clinical evaluation. Clin. Microbiol. Rev. 33:e00100. doi: 10.1128/CMR.00100-19, PMID: 31666281 PMC6822991

[ref46] Seemann T. (2015). *Snippy: Fast bacterial variant calling from NGS reads*.

[ref47] Somerville W. Thibert L. Schwartzman K. Behr M. A. (2005). Extraction of *Mycobacterium tuberculosis* DNA: a question of containment. J. Clin. Microbiol. 43, 2996–2997. doi: 10.1128/JCM.43.6.2996-2997.2005, PMID: 15956443 PMC1151963

[ref48] Talarico S. Cave M. D. Marrs C. F. Foxman B. Zhang L. Yang Z. (2005). Variation of the *Mycobacterium tuberculosis* PE_PGRS33 gene among clinical isolates. J. Clin. Microbiol. 43, 4954–4960. doi: 10.1128/JCM.43.10.4954-4960.2005, PMID: 16207947 PMC1248487

[ref49] Talarico S. Zhang L. Marrs C. F. Foxman B. Cave M. D. Brennan M. J.. (2008). *Mycobacterium tuberculosis* PE_PGRS16 and PE_PGRS26 genetic polymorphism among clinical isolates. Tuberculosis 88, 283–294. doi: 10.1016/j.tube.2008.01.001, PMID: 18313360 PMC2562508

[ref50] Tatusova T. Dicuccio M. Badretdin A. Chetvernin V. Nawrocki E. P. Zaslavsky L.. (2016). NCBI prokaryotic genome annotation pipeline. Nucleic Acids Res. 44, 6614–6624. doi: 10.1093/nar/gkw569, PMID: 27342282 PMC5001611

[ref51] Tientcheu L. D. Haks M. C. Agbla S. C. Sutherland J. S. Adetifa I. M. Donkor S.. (2016). Host immune responses differ between *M. africanum*-and *M. tuberculosis*-infected patients following standard anti-tuberculosis treatment. PLoS Negl. Trop. Dis. 10:e0004701. doi: 10.1371/journal.pntd.0004701, PMID: 27192147 PMC4871581

[ref52] Tientcheu L. D. Koch A. Ndengane M. Andoseh G. Kampmann B. Wilkinson R. J. (2017). Immunological consequences of strain variation within the *Mycobacterium tuberculosis* complex. Eur. J. Immunol. 47, 432–445. doi: 10.1002/eji.201646562, PMID: 28150302 PMC5363233

[ref53] Tundup S. Pathak N. Ramanadham M. Mukhopadhyay S. Murthy K. J. R. Ehtesham N. Z.. (2008). The co-Operonic PE25/PPE41 protein complex of *Mycobacterium tuberculosis* elicits increased humoral and cell mediated immune response. PLoS One 3:e3586. doi: 10.1371/journal.pone.0003586, PMID: 18974870 PMC2570489

[ref54] Walker B. J. Abeel T. Shea T. Priest M. Abouelliel A. Sakthikumar S.. (2014). Pilon: an integrated tool for comprehensive microbial variant detection and genome assembly improvement. PLoS One 9:e112963. doi: 10.1371/journal.pone.0112963, PMID: 25409509 PMC4237348

[ref55] World Health Organization. (2021). Global Tuberculosis Report. Geneva: World Health Organization.

[ref56] Xie J. Zhou F. Xu G. Mai G. Hu J. Wang G.. (2014). Genome-wide screening of pathogenicity islands in *Mycobacterium tuberculosis* based on the genomic barcode visualization. Mol. Biol. Rep. 41, 5883–5889. doi: 10.1007/s11033-014-3463-4, PMID: 25108673

[ref57] Yesilkaya H. Dale J. W. Strachan N. J. C. Forbes K. J. (2005). Natural transposon mutagenesis of clinical isolates of *Mycobacterium tuberculosis*: how many genes does a pathogen need? J. Bacteriol. 187, 6726–6732. doi: 10.1128/JB.187.19.6726-6732.2005, PMID: 16166535 PMC1251597

[ref58] Zhang Z. Schwartz S. Wagner L. Miller W. (2000). A greedy algorithm for aligning DNA sequences. J. Comput. Biol. 7, 203–214. doi: 10.1089/10665270050081478 10890397

