# Supplementary material for: Functional genetic variation in pe/ppe genes contributes to diversity in Mycobacterium tuberculosis lineages and potential interactions with the human host
Source: Front Microbiol. 2023 Oct 9;14:1244319. doi: 10.3389/fmicb.2023.1244319 (PMC10591178; doi:10.3389/fmicb.2023.1244319)
Supplement: Supplementary file 1 [file Data_Sheet_1.pdf]

Table S1. Metadata of samples analysed

Sample ID	Lineage	Sub-lineage	# Contigs	Length	Accession number	Sequencing technology	Assembly	Comments
**kurono**	4	4.9	1	4415078	AP014573	PacBio	HGAP2	No Illumina data
**CHIN_F1 (H37Rv)**	4	4.9	1	4415075	CP010329	PacBio	HGAP3	Illumina data (SRR3647351), corrected with Pilon
**CHIN_F28 (H37Ra)**	4	4.9	1	4421998	CP010330	PacBio	HGAP3	Illumina data (SRR3647352), corrected with Pilon
**WMB602**	4	4.9	2	4432700	ERR3427700	PacBio	Flye	Illumina data (ERR221595), corrected with Pilon
**WMB600**	4	4.8	1	4393237	ERR3427698	PacBio	Flye	Illumina data (ERR216945), corrected with Pilon
**WMU002**	4	4.7	1	4398930	ERS16427351	PacBio	Flye	Illumina data (ERR163993), corrected with Pilon
**WMU007**	4	4.6.1.2	1	4404359	ERS16427352	PacBio	Flye	Illumina data (ERR216919), corrected with Pilon
**CHIN_22115**	4	4.5	1	4401829	CP010337	PacBio	HGAP3	Illumina data (SRR3647361), corrected with Pilon
**MTB1**	4	4.5	1	4433542	CP020381	PacBio	HGAP2	No Illumina data
**H107**	4	4.5	1	4418796	CP019612	PacBio	HGAP2	No Illumina data
**CHIN_37004**	4	4.4.2	1	4417090	CP010338	PacBio	HGAP3	Illumina data (SRR3647362), corrected with Pilon
**NZ_L**	4	4.4.1.1	1	4416671	CP044345	PacBio, Illumina	Canu	Illumina data used in assembly
**WBB446**	4	4.3.4.2	1	4369979	ERR2322023	PacBio	Flye	No Illumina data
**WMU011**	4	4.3.4.2.1	1	4363273	ERS16427353	PacBio	Flye	Illumina data (ERR181979), corrected with Pilon
**WMU004**	4	4.3.4.2.1	1	4366577	ERS16427354	PacBio	Flye	No Illumina data
**WMU006**	4	4.3.4.1	1	4374435	ERS16427355	PacBio	Flye	Illumina data (ERR163992), corrected with Pilon
**ncgm946k2**	4	4.3.4.1	1	4380602	AP017901	PacBio	minimus2	No Illumina data
**WMB613**	4	4.3.3	2	4404702	ERR3427703	PacBio	Flye	No Illumina data
**WMB588**	4	4.3.3	8	4401217	ERR3427693	PacBio	Flye	Illumina data (ERR181745), corrected with Pilon
**CHIN_22103**	4	4.2.2	1	4399422	CP010339	PacBio	HGAP3	Illumina data (SRR3647353), corrected with Pilon
**MT0080**	4	4.1.2	1	4426525	CP041207	PacBio	Canu	No Illumina data
**WMB589**	4	4.1.2	1	4424878	ERR3427694	PacBio	Flye	Illumina data (ERR181717), corrected with Pilon
**H83**	4	4.1.2.1	1	4413214	CP019611	PacBio	HGAP2	No Illumina data
**WBB1452**	4	4.1.2.1	1	4416367	ERR2322019	PacBio	Flye	No Illumina data

**WMB586**	4	4.1.1.3	1	4400674	ERR3427693	PacBio	Flye	Illumina data (ERR181742), corrected with Pilon
**WMB621**	4	4.1.1.3	3	4419731	ERR3427706	PacBio	Flye	Illumina data (ERR216982), corrected with Pilon
**CHIN_2279**	2	2.2.1	1	4405033	CP010336	PacBio	HGAP3	Illumina data (SRR3647360), corrected with Pilon
**bl35049**	2	2.2.1	1	4427062	CP017593	PacBio, Illumina	Canu/Pilon	Illumina data used in assembly
**bl36918**	2	2.2.1	1	4441591	CP017594	PacBio, Illumina	Canu/Pilon	Illumina data used in assembly
**bl38774**	2	2.2.1	1	4431885	CP017595	PacBio, Illumina	Canu/Pilon	Illumina data used in assembly
**b391**	2	2.2.1	1	4406925	CP017596	PacBio, Illumina	Canu/Pilon	Illumina data used in assembly
**bl50148**	2	2.2.1	1	4444417	CP017597	PacBio, Illumina	Canu/Pilon	Illumina data used in assembly
**bl1104**	2	2.2.1	1	4380156	CP017598	PacBio, Illumina	Canu/Pilon	Illumina data used in assembly
**H54**	2	2.2.1	1	4416938	CP019610	PacBio	HGAP2	No Illumina data
**H112**	2	2.2.1	1	4406346	CP019613	PacBio	HGAP2	No Illumina data
**WC078**	2	2.2.1	1	4413712	CP022577	PacBio	HGAP2	No Illumina data
**WC059**	2	2.2.1	1	4413669	CP022578	PacBio	HGAP2	No Illumina data
**HN205**	2	2.2.1	1	4411033	AP018034	PacBio	HGAP3	No Illumina data
**HN321**	2	2.2.1	1	4421540	AP018035	PacBio	HGAP3	No Illumina data
**HN506**	2	2.2.1	1	4413362	AP018036	PacBio	HGAP3	No Illumina data
**WBB1456**	2	2.2.1	1	4409920	ERR2322017	PacBio	Flye	No Illumina data
**WBB445**	2	2.2.1	1	4410526	ERR2322022	PacBio	Flye	No Illumina data
**WMU008**	2	2.2.1	1	4418906	ERS16427356	PacBio	Flye	Illumina data (ERR245831), corrected with Pilon
**WMU005**	2	2.2.1	1	4421515	ERS16427357	PacBio	Flye	Illumina data (ERR181965), corrected with Pilon
**TB282**	2	2.2.1.2	1	4425860	CP017920	PacBio	HGAP2	No Illumina data
**MTB2**	2	2.2.2	1	4417716	CP022014	PacBio	HGAP2	No Illumina data
**CHIN_26105**	3	3	1	4426920	CP010340	PacBio	HGAP3	Illumina data (SRR3647354), corrected with Pilon
**usa750**	3	3	1	4434666	CP046309	PacBio, Illumina	HGAP3	Illumina data used in assembly
**WMU009**	3	3	1	4441198	ERS16427358	PacBio	Flye	Illumina data (ERR190402), corrected with Pilon
**WMU001**	3	3.1.1	1	4426849	ERS16427359	PacBio	Flye	Illumina data (ERR212147), corrected with Pilon
**WMU010**	3	3.1.1	1	4423118	ERS16427360	PacBio	Flye	Illumina data (ERR212002), corrected with Pilon
**usa751**	1	1	1	4441988	CP046308	PacBio, Illumina	Canu	Illumina data used in assembly

**hn24**	1	1.1.1	1	4399916	AP018033	PacBio, Illumina	HGAP3	Illumina data used in assembly
**aus**	1	1.1.1	1	4414769	CP045962	PacBio, Illumina	Canu	Illumina data (SRR10520175), corrected with Pilon
**WMB597**	1	1.1.2	1	4427144	ERR3427696	PacBio	Flye	Illumina data (ERR181798), corrected with Pilon
**WMB615**	1	1.1.2	1	4436831	ERR3427705	PacBio	Flye	Illumina data (ERR212157), corrected with Pilon
**WBB1007**	1	1.1.3	1	4432578	ERR2322014	PacBio	Flye	No Illumina data
**WBB1008**	1	1.1.3	1	4432521	ERR2322013	PacBio	Flye	No Illumina data
**WBB1009**	1	1.1.3	1	4422821	ERR2322015	PacBio	Flye	No Illumina data
**WMB614**	1	1.2.2	1	4427580	ERR3427704	PacBio	Flye	Illumina data (ERR212155), corrected with Pilon
**WMB607**	1	1.2.2	3	4415876	ERR3427701	PacBio	Flye	Illumina data (ERR221596), corrected with Pilon
**WMB596**	1	1.2.2	3	4456111	ERR3427695	PacBio	Flye	Illumina data (ERR181794), corrected with Pilon
**WBB1453**	5	5	1	4424589	ERR2322016	PacBio	Flye	No Illumina data
**WBB1454**	5	5	1	4419154	ERR2322024	PacBio	Flye	No Illumina data
**ma25**	6	6	1	4386422	CP010334	PacBio	HGAP3	Illumina data (SRR3647358), corrected with Pilon
**WBB1451**	6	6	1	4373719	ERR2322028	PacBio	Flye	No Illumina data
**WBB1457**	6	6	1	4389577	ERR2322027	PacBio	Flye	No Illumina data
**WBB1458**	6	6	1	4358247	ERR2322021	PacBio	Flye	No Illumina data
**WBB447**	6	6	1	4382892	ERR2322025	PacBio	Flye	No Illumina data
**WBB1459**	6	6	2	4385170	ERR2322020	PacBio	Flye	No Illumina data
**WBB1460**	6	6	2	4400942	ERR2322026	PacBio	Flye	No Illumina data
**bcg26**	*bovis*	*bovis*	1	4351313	CP010331	PacBio	HGAP3	Illumina data (SRR3647355), corrected with Pilon
**CP048071**	8	8	1	4379910	CP048071	PacBio	Canu	Illumina data used in assembly

Table S2. Statistical significance of differences in SNP and indel diversity between each gene functional category and *pe*/*ppe* genes.

Functional Group	π				*dxy*			
	SNPs		Indels		SNPs		Indels	
	diff*	p adj	diff*	p adj	diff*	p adj	diff*	p adj
**Cell wall and cell processes**	$1.3x10^{-4}$	**0.00518**	$3x10^{-4}$	**0**	$2.9x10^{-4}$	**1.39E-05**	$3.2x10^{-4}$	**0**
**Regulatory proteins**	$9.9x10^{-5}$	0.40263	$2.9x10^{-4}$	**2.03E-07**	$2.6x10^{-4}$	0.01723	$3.5x10^{-4}$	**5.77E-07**
**Virulence detoxification adaptation**	$1.2x10^{-4}$	0.10995	$2.7x10^{-4}$	**7.83E-06**	$2.9x10^{-4}$	**0.00136**	$3.2x10^{-4}$	**2.13E-05**
**Conserved hypotheticals**	$1.2x10^{-4}$	0.01087	$2.8x10^{-4}$	**0**	$3.1x10^{-4}$	**2.56E-06**	$3x10^{-4}$	**1.40E-11**
**Information pathways**	$1.7x10^{-4}$	**0.00171**	$3.4x10^{-4}$	**1.43E-10**	$3.9x10^{-4}$	**1.01E-06**	$4x10^{-4}$	**2.52E-09**
**Insertion seqs and phages**	$8.4x10^{-5}$	0.87033	$5.9x10^{-5}$	0.96167	$8.8x10^{-5}$	0.99593	$5.1x10^{-5}$	0.99689
**Intermediary metabolism and respiration**	$1.8x10^{-4}$	**2.44E-06**	$3.5x10^{-4}$	**0**	$3.9x10^{-4}$	**8.10E-09**	$4x10^{-4}$	**0**
**Lipid metabolism**	$1.9x10^{-4}$	**0.00010**	$3.4x10^{-4}$	**0**	$4.1x10^{-4}$	**6.60E-08**	$3.7x10^{-4}$	**2.37E-11**
**Unknown**	$-9.6x10^{-5}$	0.99921	$2.5x10^{-4}$	0.46870	$3.2x10^{-5}$	1	$2.3x10^{-4}$	0.83652

*diff = difference between mean π in *pe*/*ppe* and the other functional group of comparison or *dxy* between ancient and modern lineages in *pe*/*ppe* and the other functional groups; p adj = P-value adjusted for multiple comparisons using Tukey's Honest Significant Differences. In bold, statistically significant adjusted P values (p adj < 0.01); seqs sequences

Table S3. Classification and diversity of *pe* genes

Gene (locus)	Sub-family *	Pfam Domains	Class **	Comments	# SNPs	SNPs π	# Indels	Indels π	*dN*/*dS*
pe1 (*Rv0151c*)	V	PE, PE-PPE	S	Truncated in L1.1.3 (852_853ins)	14	$6.1x10^{-4}$	1	$3.1x10^{-5}$	4.5773
pe2 (*Rv0152c*)	V	PE, PE-PPE	C	Truncated in L8 (184_185insC)	8	$2.9x10^{-4}$	2	$3.5x10^{-5}$	0.3512
pe3 (*Rv0159c*)	V	PE, PE-PPE	C		7	$3.2x10^{-4}$	0	NA	28.8236
pe4 (*Rv0160c*)	V	PE, PE-PPE	C		7	$4.7x10^{-4}$	0	NA	26.5716
pe5 (*Rv0285*)	II	PE	C		2	$1.8x10^{-4}$	0	NA	25.418
pe6 (*Rv0335c*)	V	PE	S	Truncated in L1 (139_139del)	2	$4x10^{-4}$	1	$5.1x10^{-4}$	21.2085
pe7 (*Rv0916c*)	IV	PE	C		1	$9.3x10^{-5}$	0	NA	17.325
pe8 (*Rv1040c*)	IV	PE, PPE-SVP	C		5	$3.3x10^{-4}$	1	$3.4x10^{-5}$	0.4109
pe9 (*Rv1088*)	V	PE	C		0	NA	0	NA	0.9251
pe10 (*Rv1089*)	V	-	S	Delayed STOP in L2 and L3 (337_337del, 26 residues more)	3	$5.1x10^{-4}$	1	$1.3x10^{-3}$	16.9303
lipX/*pe11* (*Rv1169c*)	IV	PE	C		1	$9.2x10^{-5}$	0	NA	22.3073
pe12 (*Rv1172c*)	V	PE	C		5	$3.7x10^{-4}$	0	NA	1.2589
pe13 (*Rv1195*)	IV	PE	C		0	NA	0	NA	0.9251
pe14 (*Rv1214c*)	V	PE	C		3	$1.4x10^{-3}$	0	NA	0.7575
pe15 (*Rv1386*)	II	PE	C	Truncated in L8	1	$1.8x10^{-4}$	0	NA	14.0187
pe16 (*Rv1430*)	V	PE, PE-PPE	C		3	$3.1x10^{-4}$	0	NA	0.6162
pe17 (*Rv1646*)	V	PE	C		3	$2x10^{-4}$	1	$2.9x10^{-5}$	23.4983
pe18 (*Rv1788*)	IV	PE	S	Deleted in L8 and some other samples	2	$3.5x10^{-4}$	1	$2.7x10^{-4}$	0.2942
pe19 (*Rv1791*)	IV	PE	C		0	NA	0	NA	0.9251
pe20 (*Rv1806*)	IV	PE	C		3	$2.8x10^{-4}$	0	NA	0.7106
pe21 (*Rv2099c*)	V	PE	C	Pseudogene (no stop codon), continues into PE_PGRS36	2	$4.7x10^{-4}$	0	NA	22.5983

pe22 (*Rv2107*)	III	PE	C		0	NA	0	NA	0.9251
pe23 (*Rv2328*)	V	PE	C		2	7.5x10^{-4}	0	NA	26.3814
pe24 (*Rv2408*)	V	PE	C		2	6x10^{-4}	0	NA	12.4811
pe25 (*Rv2431c*)	III	PE	C		1	9.3x10^{-5}	0	NA	0
pe26 (*Rv2519*)	V	PE	C		8	4.9x10^{-4}	0	NA	0.3857
pe27 (*Rv2769c*)	IV	PE	C	Truncated in L8 (146_147insA)	6	1.2x10^{-3}	1	3.3x10^{-5}	28.4766
pe27a (*Rv3018A*)	V	-	S	Deleted in some samples, L8 delayed STOP leading to 76 residues more	1	2.6x10^{-4}	2	4.5x10^{-3}	26.2812
esxS/*pe28* (*Rv3020c*)	V	WXG100	S	Deleted in some samples	0	NA	1	1.1x10^{-3}	1
pe29 (*Rv3022A*)	V	PE	C		3	5.9x10^{-4}	0	NA	8.3594
pe31 (*Rv3477*)	IV	PE	S	Truncated in sporadic samples	3	4.5x10^{-4}	0	NA	17.5541
pe32 (*Rv3622c*)	IV	PE	S	Deleted in L6 and *bovis* (RD8)	0	NA	0	NA	1.002
pe33 (*Rv3650*)	V	PE	C		2	1.9x10^{-4}	1	9.8x10^{-5}	0.4656
pe34 (*Rv3746c*)	I	PE	C		1	8.3x10^{-5}	0	NA	10.8427
pe35 (*Rv3872*)	I	PE	S	Truncated in L5/8 (5_5del), deleted in *bovis* (RD1)	1	3.5x10^{-4}	1	1.8x10^{-4}	0.9246
pe36 (*Rv3893c*)	III	PE	C		1	1.9x10^{-4}	1	1.9x10^{-5}	28.5633
pe_pgrs1 (*Rv0109*)	V	PE	C		3	2.1x10^{-4}	1	1.9x10^{-5}	18.0182
pe_pgrs2 (*Rv0124*)	V	PE	S	Deleted in L6 (RD701), truncated in L4.3.3 (591_591insG) and L8 (481_535del)	19	5x10^{-4}	17	6.8x10^{-4}	0.8504
pe_pgrs3 (*Rv0278c*)	V	PE	S	Gene fusion with PE_PGRS4 in L2 due to deletion, duplication of PE_PGRS3 in other lineages (except H37Rv/Ra/4.6)	18	1.3x10^{-4}	26	8.5x10^{-4}	0.7585
pe_pgrs4 (*Rv0279c*)	V	PE	S	Gene fusion with PE_PGRS3 in L2 due to deletion, sporadic premature STOPs	71	1.1x10^{-3}	20	4.8x10^{-4}	0.4797
pe_pgrs5 (*Rv0297*)	V	PE	C		10	3.4x10^{-4}	7	4.5x10^{-4}	1.5899
pe_pgrs6 (*Rv0532*)	V	PE	S	Truncated in ancient lineages, L8 and *bovis* (1557_1558insT)	15	4.1x10^{-4}	15	1.1x10^{-3}	1.6463
pe_pgrs7 (*Rv0578c*)	V	PE	C		24	4.8x10^{-4}	12	3.5x10^{-4}	0.8156
pe_pgrs8 (*Rv0742*)	V	PE	C		1	5.3x10^{-5}	1	4.9x10^{-4}	19.5189
pe_pgrs9 (*Rv0746*)	V	PE	S	Truncated in sporadic samples	23	7.7x10^{-5}	30	1.2x10^{-3}	0.4987
pe_pgrs10 (*Rv0747*)	V	PE	S	Truncated in L5 (1742_1824del), *bovis*/L8 and sporadic samples	20	7.9x10^{-5}	22	1.1x10^{-3}	0.4029

pe_pgrs11 (*Rv0754*)	V	PE, His_Phos_1	C		7	$2.6x10^{-4}$	1	$1x10^{-4}$	0.4889
pe_pgrs12 (*Rv0832*)	V	PE	S	Gene fusion with PE_PGRS13 in ancient lineages (392_393insG)	1	$6.7x10^{-5}$	1	$1x10^{-3}$	0
pe_pgrs13 (*Rv0833*)	V	-	S	Gene fusion with PE_PGRS12 in ancient lineages, truncated in some L2/8 and sporadic samples	14	$3x10^{-4}$	29	$1.1x10^{-3}$	0.5459
pe_pgrs14 (*Rv0834c*)	V	PE	S	Truncated in L1.1.3 (472_472del) and sporadic samples	14	$4.5x10^{-4}$	12	$2.2x10^{-4}$	0.3931
pe_pgrs15 (*Rv0872c*)	V	PE	S	Truncated in some L2 samples (589_589del) and L8	7	$3.4x10^{-4}$	6	$1.6x10^{-4}$	0.362
pe_pgrs16 (*Rv0977*)	V	PE	S	Truncated in L4.1 (1968_1969insG)	9	$1.2x10^{-4}$	12	$2x10^{-4}$	0.2054
pe_pgrs17 (*Rv0978c*)	V	PE, NHL	K	Differences in sequence in lab strains (H37Rv and H37Ra)	13	$2.5x10^{-3}$	3	$1.4x10^{-4}$	0.2417
pe_pgrs18 (*Rv0980c*)	V	PE, NHL	K	Differences in sequence in L4.1	17	$2.6x10^{-3}$	4	$2.9x10^{-4}$	0.2674
pe_pgrs19 (*Rv1067c*)	V	PE	S	Gene fusion with PE_PGRS20 in L1 due to deletion, truncated in sporadic samples	25	$3.7x10^{-4}$	23	$1x10^{-3}$	0.8365
pe_pgrs20 (*Rv1068c*)	V	PE	S	Gene fusion with PE_PGRS19 in L1 due to deletion, in-frame insertions in L5/6/8 leading to extra PGRS motifs, truncated in sporadic samples	17	$4.5x10^{-4}$	15	$1.4x10^{-3}$	0.2407
pe_pgrs21 (*Rv1087*)	V	PE	K	Differences in sequence in L3	15	$4x10^{-4}$	35	$1.5x10^{-3}$	1.5651
pe_pgrs22 (*Rv1091*)	V	PE	S	Truncated in L1.1.3 (Q68*) and L5 (409_409del) and L8	27	$7.7x10^{-4}$	22	$6.6x10^{-4}$	0.4182
pe_pgrs23 (*Rv1243c*)	V	PE	S	Truncated in L3 (661_661del)	4	$7.9x10^{-5}$	7	$3.9x10^{-4}$	0.9196
pe_pgrs24 (*Rv1325c*)	V	PE	C	Truncated in L8	14	$5.4x10^{-4}$	6	$3x10^{-4}$	0.587
pe_pgrs25 (*Rv1396c*)	V	PE	S	Truncated in some L2 and L4, different fs	15	$9.8x10^{-4}$	8	$3.6x10^{-4}$	0.7405
pe_pgrs26 (*Rv1441c*)	V	PE	C		11	$5.6x10^{-4}$	15	$7.2x10^{-4}$	1.9764
pe_pgrs27 (*Rv1450c*)	V	PE	S	Truncated in some samples, different sequences	53	$8.5x10^{-4}$	36	$6.4x10^{-4}$	0.2947
pe_pgrs28 (*Rv1452c*)	V	PE	S	Different sequences, truncated in L5/8	131	$2x10^{-5}$	19	$1.4x10^{-3}$	0.4814
pe_pgrs29 (*Rv1468c*)	V	PE	C		5	$5.2x10^{-4}$	2	$9.8x10^{-5}$	0.5473
pe_pgrs30 (*Rv1651c*)	V	PE	C	Truncated in L8 (1345del)	15	$3.4x10^{-4}$	8	$2.2x10^{-4}$	0.7663
wag22 (*Rv1759c*)	V	-	S	Deleted in several samples (RD152)	12	$1.7x10^{-4}$	11	$4x10^{-4}$	0.4067
pe_pgrs31 (*Rv1768*)	V	PE	C		9	$1.5x10^{-4}$	3	$2.7x10^{-4}$	40.1193
pe_pgrs32 (*Rv1803c*)	V	PE	S	Truncated in sporadic samples	13	$4.2x10^{-4}$	1	$1.5x10^{-5}$	2.5034
pe_pgrs33 (*Rv1818c*)	V	PE	S	Truncated in L1 (1009_1009del)	9	$4.9x10^{-4}$	8	$7.6x10^{-4}$	0.8811
pe_pgrs34 (*Rv1840c*)	V	PE	C		1	$1.8x10^{-5}$	2	$5.3x10^{-5}$	49.3495

pe_pgrs35 (*Rv1983*)	V	PE	S	Missing in sporadic samples, truncated in L8	9	2.5x10^{-4}	1	1.7x10^{-5}	0.7638
pe_pgrs36 (*Rv2098c*)	V	PE	S	Pseudogene (no start codon), continuation of PE21 ORF in all non-L4 and L4.4 (4_5insC) leading to gene fusion PE21/PE_PGRS36	5	1.3x10^{-4}	5	5x10^{-4}	1.0747
pe_pgrs37 (*Rv2126c*)	V	-	C		3	1.4x10^{-4}	2	7.2x10^{-5}	0.3612
pe_pgrs38 (*Rv2162c*)	V	PE	C		11	4.9x10^{-4}	8	4x10^{-4}	0.6228
pe_pgrs39 (*Rv2340c*)	V	PE	C		10	4.9x10^{-4}	0	NA	0.6159
pe_pgrs40 (*Rv2371*)	V	PE	C		2	1.1x10^{-3}	0	NA	0
pe_pgrs41 (*Rv2396*)	V	PE	S	Truncated in L3.1.1 (397_397del)	10	7.7x10^{-4}	3	1x10^{-4}	0.3329
pe_pgrs42 (*Rv2487c*)	V	PE	S	Truncated in 2 L4 samples	11	3.1x10^{-4}	4	1.4x10^{-4}	0.4927
pe_pgrs43 (*Rv2490c*)	V	PE	C	L8 truncated	24	2.5x10^{-4}	13	1.3x10^{-4}	1.2423
pe_pgrs44 (*Rv2591*)	V	PE	C		11	1x10^{-3}	8	4.1x10^{-4}	0.4302
pe_pgrs45 (*Rv2615c*)	V	PE	K	Differences in sequence	20	2.3x10^{-3}	4	4.2x10^{-4}	0.4864
pe_pgrs46 (*Rv2634c*)	V	PE	S	Truncated in L5 (1490_1491insG) and sporadic samples	17	5.7x10^{-4}	12	1.9x10^{-4}	2.0905
pe_pgrs47 (*Rv2741*)	V	PE	S	Truncated in L6 and *bovis* (28_28del)	12	7.6x10^{-4}	5	2.1x10^{-4}	0.2756
pe_pgrs48 (*Rv2853*)	V	PE	S	Sequences missing/deleted	17	5.4x10^{-4}	4	8.9x10^{-5}	1.5105
lipY (*Rv3097c*)	V	PE, Abhydrolase_3	S	Truncated in sporadic samples	10	6.6x10^{-4}	3	6.3x10^{-5}	1.5359
pe_pgrs49 (*Rv3344c*)	V	-	S	Change in ORF in all except L4 (20_20del) making it continuation of PE_PGRS50 (gene fusion)	9	2.9x10^{-4}	10	8.8x10^{-4}	2.3838
pe_pgrs50 (*Rv3345c*)	V	PE	S	Truncated in L1 and some L2 (811_811del); rest of L2/3/5/6/8/*bovis* ORF continues into PE_PGRS49 (4356_4356del = PE_PGRS49 20_20del) leading to gene fusion	35	1x10^{-4}	50	1.2x10^{-3}	1.1135
pe_pgrs51 (*Rv3367*)	V	PE	S	Truncated in L5 (309_391del) and sporadic samples	15	3.3x10^{-4}	4	1.2x10^{-4}	0.8626
pe_pgrs52 (*Rv3388*)	V	PE	S	Truncated in sporadic samples	9	1.5x10^{-4}	16	8.4x10^{-4}	0.6388
pe_pgrs53 (*Rv3507*)	V	PE	S	Truncated in L5 and some L2samples (1111_1111del)	29	4.6x10^{-4}	39	9x10^{-4}	0.8128
pe_pgrs54 (*Rv3508*)	V	PE	S	Truncated in L6 (461_462insC), some L3 (3718_1718del) and sporadic samples (*bovis*/L8)	422	4.6x10^{-5}	97	1.6x10^{-3}	0.7237
pe_pgrs55 (*Rv3511*)	V	PE	S	Truncated in L5 (1213_1213del), rest except 4.7-9 ORF continues into PE_PGRS56 (2108_2108del) leading to gene fusion	13	3.3x10^{-4}	38	1.4x10^{-3}	0.9282

pe_pgrs56 (*Rv3512*)	V	-	S	Truncated in L5, continuation of PE_PGRS55 in the rest except L4.7-9 (1_1del = PE_PGRS55 2108_2108del) leading to gene fusion	48	$3.6x10^{-5}$	55	$1.6x10^{-3}$	0.9011
pe_pgrs57 (*Rv3514*)	V	PE	S	Truncated in L6 (461_462insC), truncated in most of L2 (796_850del) and in sporadic samples, deleted in L5/8 and sporadic samples	687	$3.4x10^{-5}$	208	$3.1x10^{-3}$	0.6838
pe_pgrs58 (*Rv3590c*)	V	PE	C	L8 different truncated due to different sequence	12	$7x10^{-4}$	5	$1.6x10^{-4}$	1.7062
pe_pgrs59 (*Rv3595c*)	V	PE	C		7	$3.6x10^{-4}$	4	$4.8x10^{-4}$	0.6077
pe_pgrs60 (*Rv3652*)	V	PE	S	Change in ORF in L4.3 (249_249del) leading to longer protein sequence	1	$8.2x10^{-4}$	2	$6.5x10^{-4}$	28.6189
pe_pgrs61 (*Rv3653*)	V	PE	S	Truncated in most L3 (115_115del)	5	$1.8x10^{-4}$	4	$3.2x10^{-4}$	19.6831
pe_pgrs62 (*Rv3812*)	V	PE	C		6	$3.3x10^{-4}$	1	$1.8x10^{-5}$	0.6114

* Sub-family classification based on [5].

** Class: C = conserved; S = structural variant; K = unique *k-mer* profile

Table S4. Classification and diversity of *ppe* genes

Gene (locus)	Sub-family *	Pfam Domains	Class **	Comments	# SNPS	SNPs π	# Indels	Indels π	*dN*/*dS*
ppe1 (*Rv0096*)	II (PPW)	PPE, PPE-PPW	C		10	$8x10^{-4}$	0	NA	1.1121
ppe2 (*Rv0256c*)	II (PPW)	PPE, PPE-PPW	C		10	$5.2x10^{-4}$	0	NA	2.7497
ppe3 (*Rv0280*)	II (PPW)	PPE, PPE-PPW	C		7	$7.7x10^{-4}$	3	$1.1x10^{-4}$	1.1022
ppe4 (Rv0286)	II (PPW)	PPE, PPE-PPW	C		9	$4.8x10^{-4}$	0	NA	0.1912
ppe5 (*Rv0304c*)	V (MPTR)	MPTR	S	Truncated in L5/6/*bovis* (2997_2997del) and in sporadic samples, >50% deleted in L8	34	$3.7x10^{-4}$	11	$1.6x10^{-4}$	0.4433
ppe6 (*Rv0305c*)	V (MPTR)	PPE, MPTR	S	All samples except L1.1.3 (truncated 2678_2678del) and lab strains H37Rv/Ra change in ORF (2429_2429del) which continues until the end of PPE5	7	$3.2x10^{-4}$	8	$1.3x10^{-4}$	0.42
ppe7 (*Rv0354c*)	V (MPTR)	-	C	Different from H37Rv, 42 aa longer (372_373insG)	0	NA	1	0	0.9251
ppe8 (*Rv0355c*)	V (MPTR)	PPE, MPTR	S	Truncated in some L2 (453_453del); ancient lineages change in ORF (9889_9890insTA) leading to 211 residues more (until the end of PPE7 ORF)	41	$4.1x10^{-4}$	17	$1.9x10^{-4}$	0.4087
ppe9 (*Rv0388c*)	IV (SVP)	PPE, PPE-SVP	C	Different from H37Rv, 263 aa longer and SVP domain (492_493insC, 501_502insC)	10	$5.1x10^{-5}$	2	0	1.3175
ppe10 (*Rv0442c*)	V (MPTR)	PPE, MPTR	C		9	$3.4x10^{-4}$	3	$1.4x10^{-4}$	2.0863
ppe11 (*Rv0453*)	II (PPW)	PPE, PPE-PPW	C		4	$1.8x10^{-4}$	0	NA	0.3283
ppe12 (*Rv0755c*)	V (MPTR)	PPE, MPTR	S	Truncated in L5 (87_87del)	12	$3.9x10^{-4}$	4	$7.1x10^{-5}$	0.4687
ppe13 (*Rv0878c*)	V (MPTR)	PPE, MPTR	C	polyC/polyA region masked in analysis, as there might be errors due to sequencing	10	$5.4x10^{-4}$	5	$6.1x10^{-4}$	0.5377
ppe14 (*Rv0915c*)	IV (SVP)	PPE, PPE-SVP	C		4	$1.5x10^{-4}$	0	NA	0.93
ppe15 (*Rv1039c*)	IV (SVP)	PPE, PPE-SVP	C		2	$7x10^{-5}$	0	NA	23.8303
ppe16 (*Rv1135c*)	V (MPTR)	PPE, MPTR	S	Truncated in most L2 (IS6110) and L6 (1279_1283del)	8	$2.2x10^{-4}$	2	$2.6x10^{-4}$	14.9883
ppe17 (*Rv1168c*)	IV (SVP)	PPE, PPE-SVP	C		5	$6.4x10^{-4}$	0	NA	0.4819

ppe18 (*Rv1196*)	IV (SVP)	PPE, PPE-SVP	K	Different sequences	98	$6.8x10^{-3}$	8	$5.2x10^{-4}$	0.3642
ppe19 (*Rv1361c*)	IV (SVP)	PPE, PPE-SVP	S	Truncated in L1.1.3 (Q145*)	104	$3.9x10^{-3}$	2	6.93E-05	0.4275
ppe20 (*Rv1387*)	II (PPW)	PPE, PPE-PPW	C		9	$3.8x10^{-4}$	0	NA	1.3113
ppe21 (*Rv1548c*)	V (MPTR)	PPE, MPTR	C		17	$2.5x10^{-4}$	3	$5.2x10^{-5}$	0.4219
ppe22 (*Rv1705c*)	IV (SVP)	PPE, PPE-SVP	C		6	$5.7x10^{-4}$	0	NA	27.207
ppe23 (*Rv1706c*)	IV (SVP)	PPE, PPE-SVP	C		4	$2.9x10^{-4}$	0	NA	1.0831
ppe24 (*Rv1753c*)	V (MPTR)	PPE, MPTR	S	Truncated in sporadic samples, truncated in L8 by IS*6110*	29	$5.2x10^{-4}$	10	$3.1x10^{-4}$	0.1083
ppe25 (*Rv1787*)	IV (SVP)	PPE, PPE-SVP	S	Deleted in some samples	17	$1.2x10^{-3}$	3	0.00021842	0.7069
ppe26 (*Rv1789*)	IV (SVP)	PPE, PPE-SVP	S	Deleted in some samples	9	$2.7x10^{-4}$	2	$9.2x10^{-5}$	0.3614
ppe27 (*Rv1790*)	IV (SVP)	PPE, PPE-SVP	S	Deleted in some samples	2	$2.4x10^{-4}$	1	$7.7x10^{-5}$	0.2488
ppe28 (*Rv1800*)	V (MPTR)	PPE, PE-PPE	C		9	$4.8x10^{-4}$	1	$1.4x10^{-5}$	2.5387
ppe29 (*Rv1801*)	IV (SVP)	PPE, PPE-SVP	C		14	$4.9x10^{-4}$	0	NA	0.9722
ppe30 (Rv1802)	IV (SVP)	PPE, PPE-SVP	S	Truncated in L6 (Q162*), truncated in some L2	7	$2.4x10^{-4}$	1	$3.9x10^{-5}$	1.6392
ppe31 (*Rv1807*)	IV (SVP)	PPE, PPE-SVP	C	Truncated in L8	9	$3.6x10^{-4}$	1	$2.3x10^{-5}$	0.7717
ppe32 (*Rv1808*)	IV (SVP)	PPE, PPE-SVP	C		3	$7.9x10^{-4}$	0	NA	0.2067
ppe33 (*Rv1809*)	IV (SVP)	PPE, PPE-SVP	S	L1/5/6/8 with 1 residue more (*469S), truncated in *bovis*	11	$6.1x10^{-4}$	1	$2x10^{-5}$	0.2663
ppe34 (*Rv1917c*)	V (MPTR)	PPE, MPTR	S	Truncated in most lineages due to IS6110	43	$1.4x10^{-4}$	25	$7.8x10^{-4}$	0.5102
ppe35 (*Rv1918c*)	V (MPTR)	PPE, MPTR	S	Truncated in sporadic samples	14	$3.6x10^{-4}$	2	$2.8x10^{-5}$	1.7086
ppe36 (*Rv2108*)	III	PPE	C		3	$1.5x10^{-4}$	0	NA	0.1015
ppe37 (*Rv2123*)	II (PPW)	PPE, PPE-PPW	S	Truncated in L2 (503_503del) and L3 (1219_1219del), delayed STOP in some L4 (1016_1017del) adding 23 residues)	12	$3.6x10^{-4}$	4	$4.9x10^{-4}$	0.74

ppe38 (*Rv2352c*)	IV (SVP)	PPE, PPE-SVP	S	Deletion of beginning of gene in L2 (RD185), samples missing, deleted in *bovis*/L8	2	$4.3x10^{-5}$	7	$6.6x10^{-4}$	12.0194
ppe39 (*Rv2353c*)	V (MPTR)	MPTR	S	Deletion of beginning of the gene in most isolates, missing samples, deleted in L8	4	$2.2x10^{-4}$	6	$7x10^{-4}$	0.2822
ppe40 (*Rv2356c*)	V (MPTR)	PPE, MPTR	S	Missing samples, truncated in sporadic samples (IS6110)	2	$2.9x10^{-5}$	4	$8.9x10^{-5}$	18.7541
ppe41 (*Rv2430c*)	III	PPE	C		1	$4.8x10^{-5}$	1	$4.8x10^{-5}$	0
ppe42 (*Rv2608*)	V (MPTR)	PPE, PE-PPE	C		6	$2.1x10^{-4}$	0	NA	0.2935
ppe43 (*Rv2768c*)	IV (SVP)	PPE, PPE-SVP	S	Truncated in L5 (449_454del)	5	$3.9x10^{-4}$	1	$4.6x10^{-5}$	26.114
ppe44 (*Rv2770c*)	IV (SVP)	PPE, PPE-SVP	C		8	$7.8x10^{-4}$	0	NA	0.6649
ppe45 (*Rv2892c*)	IV (SVP)	PPE, PPE-SVP	S	Truncated in L6 (W75*)	5	$3.6x10^{-4}$	0	NA	19.2502
ppe46 (*Rv3018c*)	II (PPW)	PPE, PPE-PPW	S	Truncated in 4.1.1.3 (IS6110) and in other sporadic samples	28	$2.5x10^{-3}$	4	$1.9x10^{-4}$	0.4056
ppe47 (*Rv3021c*)	II (PPW)	PPE, PPE-PPW	S	Pseudogene, all different to reference (12_13insG) making the ORF to continue until the end of PPE47; deleted in some samples	17	$6x10^{-4}$	8	$5.4x10^{-4}$	0.3328
ppe48 (*Rv3022c*)	II (PPW)	PPE	C	Pseudogene, no stop codon until end of PPE47 except in ref, where fs in PPE47 (12_13insG) creates premature stop	1	$1.6x10^{-3}$	1	0.0001143	14.7954
ppe49 (*Rv3125c*)	IV (SVP)	PPE, PPE-SVP	S	Truncated in L1.1.3 and L3.1.1 (IS6110), truncated in sporadic samples	10	$3x10^{-4}$	5	$2.3x10^{-4}$	0.5397
ppe50 (*Rv3135*)	IV (SVP)	PPE, PPE-SVP	S	L1 deleted; insertion in L2/5/6/*bovis*/L8 adding SVP domain (331_332ins)	5	$7.6x10^{-4}$	4	$2.7x10^{-3}$	0.4003
ppe51 (*Rv3136*)	IV (SVP)	PPE, PPE-SVP	C		3	$7.2x10^{-5}$	0	NA	0.1923
ppe52 (*Rv3144c*)	V (MPTR)	PPE	S	Truncated in 3.1.1 (970_970del)	8	$5.3x10^{-4}$	2	$8.9x10^{-5}$	0.4428
ppe53 (*Rv3159c*)	V (MPTR)	PPE, MPTR	S	L1/2/3/4.1/4.2/5/6 truncated (88_89ins or IS6110)	12	$5.7x10^{-4}$	4	$4x10^{-4}$	2.3535
ppe54 (*Rv3343c*)	V (MPTR)	PPE, MPTR	S	Truncated in sporadic samples (IS6110/big insertions)	127	0	21	$3.8x10^{-4}$	0.3772
ppe55 (*Rv3347c*)	V (MPTR)	PPE, MPTR	S	Truncated in L4.5 (IS6110), L5/6/*bovis* and sporadic samples, missing samples	151	$1.3x10^{-3}$	23	$1.3x10^{-4}$	0.4674
ppe56 (*Rv3350c*)	V (MPTR)	PPE, MPTR	S	Truncated in L2 (6081_6081del) and L6 (6586_6586del), missing samples	223	$3x10^{-4}$	20	$1.1x10^{-4}$	0.3403
ppe57 (*Rv3425*)	III	PPE	S	Deleted in all L1, half of L4, L8 and some other sporadic samples; truncated in L2 (226_226del)	11	$3.8x10^{-4}$	5	$1.9x10^{-3}$	0.6238

ppe58 (*Rv3426*)	III	PPE	S	Deleted in L8 and some other samples; truncated in all except L4.9 (373_373del)	6	$3.2x10^{-4}$	2	$1.1x10^{-3}$	2.2304
ppe59 (*Rv3429*)	III	PPE	S	Deleted (>50%) in sporadic samples	45	$9.5x10^{-3}$	1	$1x10^{-4}$	2.4137
ppe60 (*Rv3478*)	IV (SVP)	PPE, PPE-SVP	S	Truncated in sporadic samples	85	$4.4x10^{-3}$	5	$5.1x10^{-4}$	0.6678
ppe61 (*Rv3532*)	IV (SVP)	PPE, PPE-SVP	C		6	$3.5x10^{-4}$	3	$1.5x10^{-4}$	24.5627
ppe62 (*Rv3533c*)	V (MPTR)	PPE, MPTR	C		5	$9.4x10^{-5}$	3	$4.8x10^{-5}$	0.183
ppe63 (*Rv3539*)	V (MPTR)	PPE, PE-PPE	C		6	$3.8x10^{-4}$	0	NA	1.6317
ppe64 (*Rv3558*)	V (MPTR)	PPE, MPTR	S	Truncated in L3 (63_64del)	4	$2.1x10^{-4}$	3	$2.9x10^{-4}$	0.2259
ppe65 (*Rv3621c*)	IV (SVP)	PPE, PPE-SVP	S	Deleted in L6/*bovis* (RD8)	2	$2x10^{-4}$	0	NA	0.3229
ppe66 (*Rv3738c*)	II (PPW)	PPE, PPE-PPW	S	Deleted in L3	6	$4.4x10^{-4}$	2	$1.9x10^{-4}$	0.6127
ppe67 (*Rv3739c*)	II (PPW)	PPE	S	Truncated in L3 (152_234del); L1/5/8 delayed STOP (*78W) leading ORF to continue until the end of PPE66	5	$1.7x10^{-3}$	2	$6.8x10^{-4}$	1.1496
pep68 (*Rv3873*)	I	PPE	C	Deleted in *bovis* (RD1)	4	$3.3x10^{-4}$	0	NA	27.142
ppe69 (*Rv3892c*)	III	PPE	S	Truncated in some L2 due to deletion	9	$5.9x10^{-4}$	2	$9.1x10^{-5}$	0.6972

* Sub-family classification [5]

** Class: C = conserved; S = Structural variant; K = unique *k-mer* profile

Table S5. List of conserved genes with start and end coordinates in H37Rv reference

ID	Gene	Start	End
Rv0096	*ppe1*	105324	106715
Rv0109	*pe_pgrs1*	131382	132872
Rv0152c	*pe2*	179319	180896
Rv0159c	*pe3*	187433	188839
Rv0160c	*pe4*	188931	190439
Rv0256c	*ppe2*	307877	309547
Rv0280	*ppe3*	339364	340974
Rv0285	*pe5*	349624	349932
Rv0286	*ppe4*	349935	351476
Rv0297	*pe_pgrs5*	361334	363109
Rv0354c	*ppe7*	424269	424694
Rv0388c	*ppe9*	467459	468001
Rv0442c	*ppe10*	530751	532214
Rv0453	*ppe11*	543174	544730
Rv0578c	*pe_pgrs7*	671996	675916
Rv0742	*pe_pgrs8*	832981	833508
Rv0754	*pe_pgrs11*	846159	847913
Rv0878c	*ppe13*	976872	978203
Rv0915c	*ppe14*	1020058	1021329
Rv0916c	*pe7*	1021344	1021643
Rv1039c	*ppe15*	1161297	1162472
Rv1040c	*pe8*	1162549	1163376
Rv1088	*pe9*	1214513	1214947
Rv1168c	*ppe17*	1298764	1299804
Rv1169c	*lipX/pe11*	1299822	1300124
Rv1172c	*pe12*	1301755	1302681
Rv1195	*pe13*	1339003	1339302
Rv1214c	*pe14*	1357293	1357625
Rv1325c	*pe_pgrs24*	1488154	1489965
Rv1386	*pe15*	1561464	1561772
Rv1387	*ppe20*	1561769	1563388
Rv1430	*pe16*	1606386	1607972
Rv1441c	*pe_pgrs26*	1618209	1619684
Rv1468c	*pe_pgrs29*	1655609	1656721
Rv1548c	*ppe21*	1751297	1753333
Rv1646	*pe17*	1855764	1856696
Rv1651c	*pe_pgrs30*	1862347	1865382
Rv1705c	*ppe22*	1931497	1932654
Rv1706c	*ppe23*	1932694	1933878
Rv1768	*pe_pgrs31*	2000614	2002470

Rv1791	*pe19*	2029904	2030203
Rv1800	*ppe28*	2039453	2041420
Rv1801	*ppe29*	2042001	2043272
Rv1806	*pe20*	2048072	2048371
Rv1807	*ppe31*	2048398	2049597
Rv1808	*ppe32*	2049921	2051150
Rv1840c	*pe_pgrs34*	2087971	2089518
Rv2099c	*pe21*	2358033	2358206
Rv2107	*pe22*	2367359	2367655
Rv2108	*ppe36*	2367711	2368442
Rv2126c	*pe_pgrs37*	2387202	2387972
Rv2162c	*pe_pgrs38*	2423240	2424838
Rv2328	*pe23*	2600731	2601879
Rv2340c	*pe_pgrs39*	2617667	2618908
Rv2371	*pe_pgrs40*	2651753	2651938
Rv2408	*pe24*	2706017	2706736
Rv2430c	*ppe41*	2727336	2727920
Rv2431c	*pe25*	2727967	2728266
Rv2490c	*pe_pgrs43*	2801254	2806236
Rv2519	*pe26*	2835785	2837263
Rv2591	*pe_pgrs44*	2921551	2923182
Rv2608	*ppe42*	2935046	2936788
Rv2769c	*pe27*	3078158	3078985
Rv2770c	*ppe44*	3079309	3080457
Rv3022c	*ppe48*	3380440	3380682
Rv3022A	*pe29*	3380679	3380993
Rv3136	*ppe51*	3501794	3502936
Rv3532	*ppe61*	3969343	3970563
Rv3533c	*ppe62*	3970705	3972453
Rv3539	*ppe63*	3978059	3979498
Rv3590c	*pe_pgrs58*	4031404	4033158
Rv3595c	*pe_pgrs59*	4036731	4038050
Rv3650	*pe33*	4091233	4091517
Rv3746c	*pe34*	4196171	4196506
Rv3812	*pe_pgrs62*	4276571	4278085
Rv3873	*pep68*	4351075	4352181
Rv3893c	*pe36*	4375762	4375995

Table S6. Genes with IS*6110* integrated within the coding region

Locus	Gene	# Samples with IS*6110**	Consequence
Rv1040c	*pe8*	1	frameshift
Rv1135c	*ppe16*	14 (L2.2.1)	frameshift
Rv1753c	*ppe24*	1	frameshift
Rv1800	*ppe28*	1	frameshift
Rv1917c	*ppe34*	34 (n=20 L2, n=5 L3)	frameshift/stop codon
Rv2352c	*ppe38*	17 (L2)	Frameshift/stop codon
Rv2356c	*ppe40*	1	frameshift
Rv3018c	*ppe46*	3 (n=2 L4.1.1.3)	frameshift/stop codon
Rv3021c	*ppe47*	1	stop codon
Rv3125c	*ppe49*	7 (n=3 L3.1.1, n=2 L1.1.3)	frameshift/stop codon
Rv3159c	*ppe53*	2	frameshift/stop codon
Rv3343c	*ppe54*	1	stop codon
Rv3347c	*ppe55*	3 (L4.5)	frameshift

* In brackets, if there is lineage patterns, number, and lineage where samples belonged to.

Table S7. Lineage-specific variants.

Gene (locus)	Lineage	Variant	AF in lineage *	AF in rest **	Gene Class	Comment
ppe1 **(*Rv0096*)**	L6	P298P	0.986	0	C	
pe1 **(*Rv0151c*)**	*M. bovis*	G26R	0.972	0	S	
	L3	G369R	1	0	S	
	L6	P494L	1	0	S	
pe3 **(*Rv0159c*)**	*M. bovis*	P255T	1	0	C	
	L3	S175P	0.999	0	C	
pe4 **(*Rv0160c*)**	L1	K164N	1	0	C	
	L3	F197S	0.999	0	C	
ppe2 **(*Rv0256c*)**	L1	T412T	0.984	0	C	
	L5	E140G	1	0	C	
	L5	D431N	1	0	C	
ppe3 **(*Rv0280*)**	L5	E448D	1	0	C	
	L6	M450T	1	0	C	
ppe4 **(*Rv0286*)**	L3	L52M	0.955	0	C	
	Ancient	A185A	1	0	C	
pe_pgrs5 **(*Rv0297*)**	L1	G225D	0.952	0	C	
ppe5 **(*Rv0304c*)**	L1	I1273V	0.999	0	S	
	L3	G960A	0.955	0	S	
	Ancient	S1765F	0.998	0	S	
ppe8 **(*Rv0355c*)**	L1	139_139del	1	0	S	
	M. bovis	G2403G	1	0	S	
	L1	V118A	1	0	S	
	L3	D741N	0.983	0	S	
	L3	S1920F	0.954	0	S	
	L5	F414V	1	0	S	
	Ancient	I3250F	1	0	S	
	Ancient	9889_9890insATA	0.999	0	S	Change in ORF of PPE8 until the end of PPE7 (gene fusion)
ppe10 **(*Rv0442c*)**	*M. bovis*	W8*	0.991	0	C	Truncated protein
	L3	W147S	1	0	C	
	L6	G288A	1	0	C	
pe_pgrs6 **(*Rv0532*)**	L3	A124V	0.997	0	S	
pe_pgrs7 **(*Rv0578c*)**	L1	G951R	0.981	0	C	
	L3	G405G	0.978	0	C	
pe_pgrs10 **(*Rv0747*)**	L3	G799G	0.953	0	S	
pe_pgrs11 **(*Rv0754*)**	L1	G280R	0.999	0	C	

***ppe12* (*Rv0755c*)**	L5	G378S	0.996	0	S	
	Ancient	R545K	0.999	0	S	
***pe_pgrs14* (*Rv0834c*)**	L1	G668D	0.977	0	S	
	Ancient	A246A	0.950	0	S	
***pe_pgrs15* (*Rv0872c*)**	*M. bovis*	L113L	0.991	0	S	
***ppe13* (*Rv0878c*)**	L1	G336G	1	0	C	
	L6	N244N	0.993	0	C	
***ppe14* (*Rv0915c*)**	L5	T293M	1	0	C	
***pe_pgrs16* (*Rv0977*)**	L4.1	1968_1969insG	1	0	S	Truncated protein
***pe10* (*Rv1089*)**	L2/L3	337_337del	0.999	0	S	Delayed STOP, 26 more residues
***pe_pgrs22* (*Rv1091*)**	L2	G730G	0.952	0	S	
	L5	G118G	1	0	S	
***ppe16* (*Rv1135c*)**	L5	G349R	0.984	0	S	
	L6	1279_1283del	1	0	S	Truncated protein
***ppe17* (*Rv1168c*)**	L2	P167L	0.982	0	C	
***pe12* (*Rv1172c*)**	L5	L217F	1	0	C	
***ppe18* (*Rv1196*)**	L5	H234R	1	0	K	
***pe14* (*Rv1214c*)**	L2	A106A	0.981	0	C	
***pe_pgrs23* (*Rv1243c*)**	L5	G280G	0.952	0	S	
***pe_pgrs24* (*Rv1325c*)**	L5	L101R	1	0	C	
***ppe19* (*Rv1361c*)**	L3	F4V	0.965	0	S	
***ppe20* (*Rv1387*)**	*M. bovis*	V94A	0.991	0	C	
***pe16* (*Rv1430*)**	L2	A96A	0.998	0	C	
***pe17* (*Rv1646*)**	L3	T285I	1	0	C	
***pe_pgrs30* (*Rv1651c*)**	*M. bovis*	A172V	0.978	0	C	
	L3	T600N	0.999	0	C	
	L5	R115L	1	0	C	
***ppe23* (*Rv1706c*)**	L6	S37P	1	0	C	
***ppe24* (*Rv1753c*)**	L5	S716R	1	0	S	
***ppe25* (*Rv1787*)**	*M. bovis*	925_927del	1	0	S	In-frame
***ppe28* (*Rv1800*)**	Ancient	C144W	0.994	0	C	
***ppe29* (*Rv1801*)**	L5	A366P	0.996	0	C	

***pe_pgrs32* (*Rv1803c*)**	L5	E76D	1	0	S	
	L5	A483T	1	0	S	
***ppe31* (*Rv1807*)**	L5	H188Y	1	0	C	
***ppe33* (*Rv1809*)**	L3	G22S	0.985	0	S	
***ppe36* (*Rv2108*)**	L5	I25I	1	0	C	
***ppe37* (*Rv2123*)**	L5	V124M	1	0	S	
***pe_pgrs39* (*Rv2340c*)**	L5	A109T	1	0	C	
***pe_pgrs40* (*Rv2371*)**	L5	D29D	1	0	C	
***pe_pgrs41* (*Rv2396*)**	*M. bovis*	S26N	0.991	0	S	
***pe24* (*Rv2408*)**	L2	G216V	0.982	0	C	
***pe_pgrs42* (*Rv2487c*)**	L5	G125G	1	0	S	
***pe_pgrs43* (*Rv2490c*)**	L6	W1503R	0.971	0	C	
***pe26* (*Rv2519*)**	L3	S330L	0.955	0	C	
	L5	G160S	1	0	C	
***pe_pgrs44* (*Rv2591*)**	L5	A439A	0.984	0	C	
	Ancient	G478G	0.994	0	C	
***pe_pgrs45* (*Rv2615c*)**	L3	G437G	0.998	0	K	
***pe_pgrs47* (*Rv2741*)**	L1	S20S	1	0	S	
	Ancient	G383G	0.969	0	S	
***ppe43* (*Rv2768c*)**	L5	449_454del	0.988	0	S	Truncated protein
***ppe44* (*Rv2770c*)**	L1	G59V	1	0	C	
***ppe45* (*Rv2892c*)**	L6	W75*	1	0	S	Truncated protein
***ppe48* (*Rv3022A*)**	L3	I64L	0.999	0	C	
***lipY* (*Rv3097c*)**	L4	A58G	1	0	S	
	L5	F129S	1	0	S	
***ppe54* (*Rv3343c*)**	L3	G2189S	0.982	0	S	
***ppe56* (*Rv3350c*)**	L6	6586_6586del	1	0	S	Truncated protein
***pe_pgrs55* (*Rv3511*)**	L5	1411_1411del	0.956	0	S	Truncated protein
***ppe61* (*Rv3532*)**	L1	T257M	1	0	C	
***ppe63* (*Rv3539*)**	L1	Y365N	1	0	C	
***ppe64* (*Rv3558*)**	L1	G306S	0.998	0	S	
	L3	63_64del	0.955	0	S	Truncated protein

***pe_pgrs58* (*Rv3590c*)**	L2	A314V	0.969	0	C
***pe_pgrs59* (*Rv3595c*)**	L5	G22D	1	0	C

AF = Allele frequency; * AF in indicated lineage; ** AF in the group of samples from other lineages.

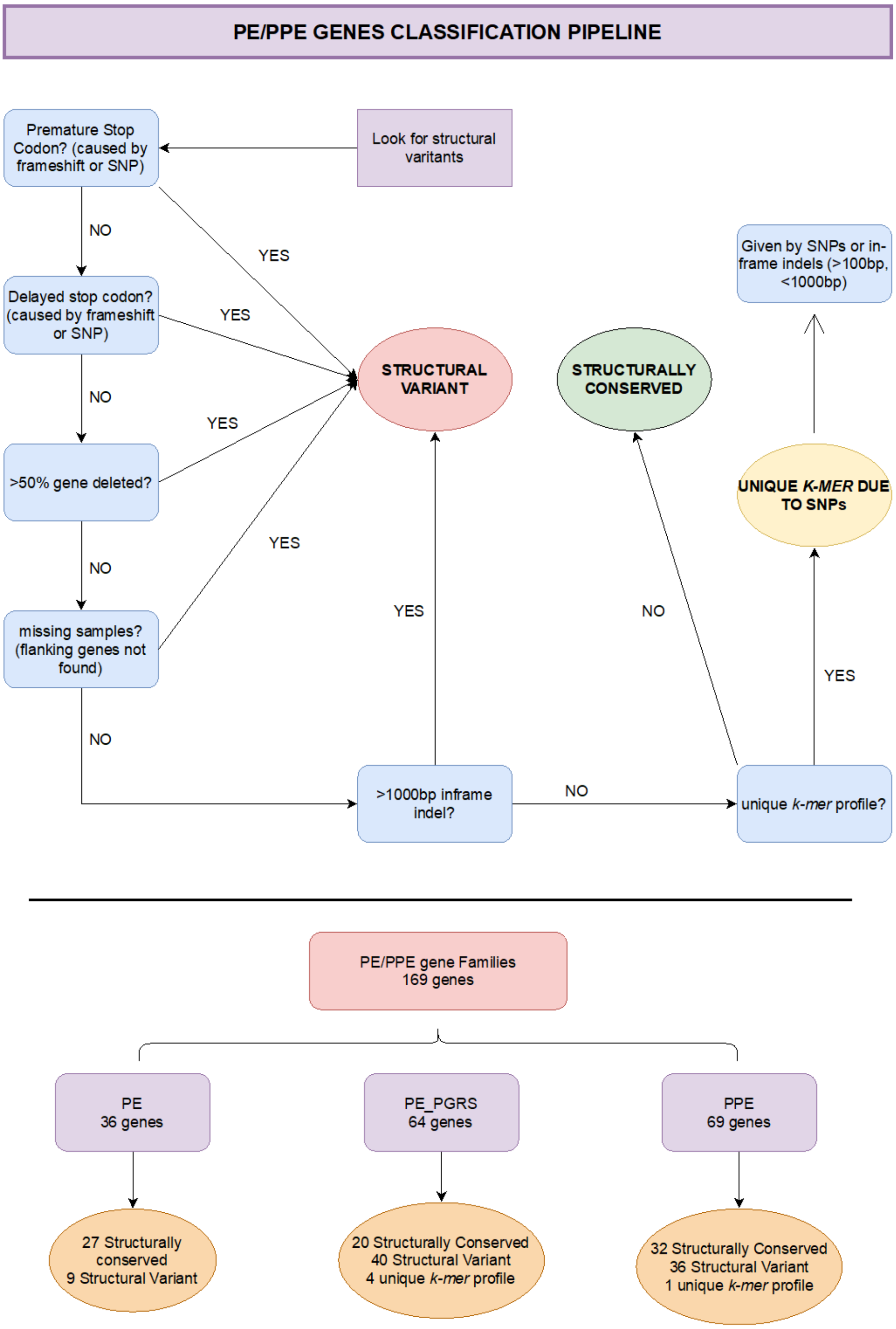

Figure S1. Flowchart showing the pipeline followed for the classification of *pe* and *ppe* genes.

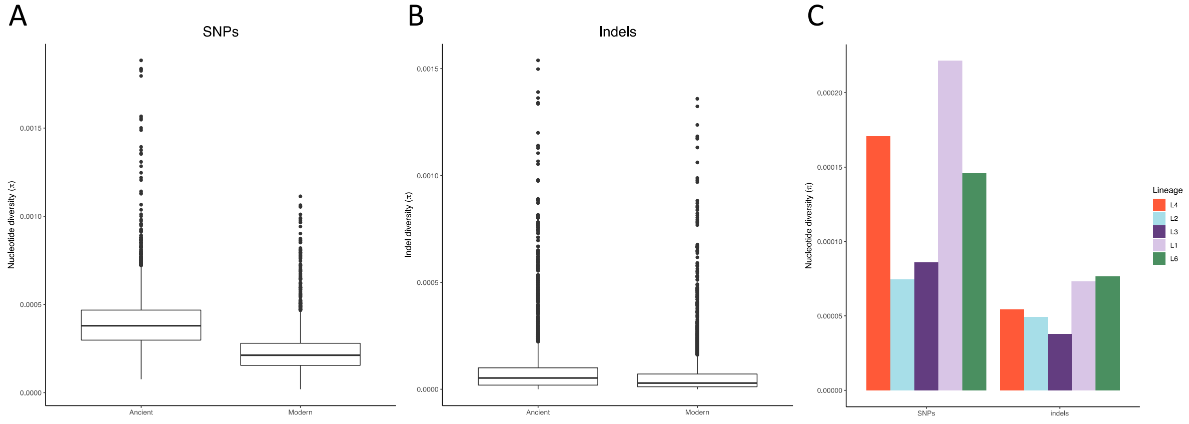

Figure S2. Boxplots of whole genome nucleotide diversity (π) for (A) SNPs and (B) indels between ancient and modern lineages. (C) SNP and indel π by lineage (L5/8 and *M. bovis* excluded due to low number of isolates).

A

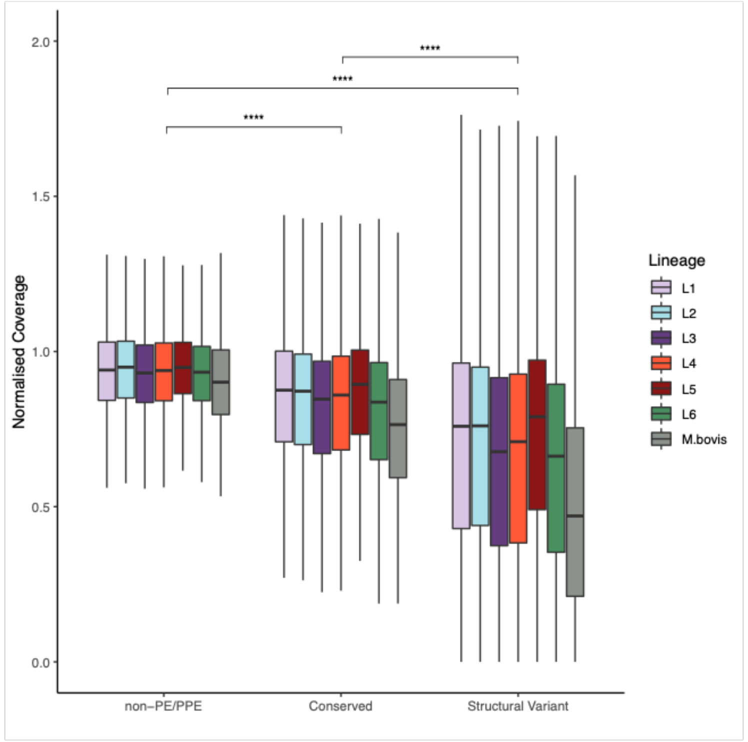

2.0
1.5
1.0
0.5
0.0
Normalised Coverage
****
****
****
non−PE/PPE
Conserved
Structural Variant
Lineage
L1
L2
L3
L4
L5
L6
M.bovis

B

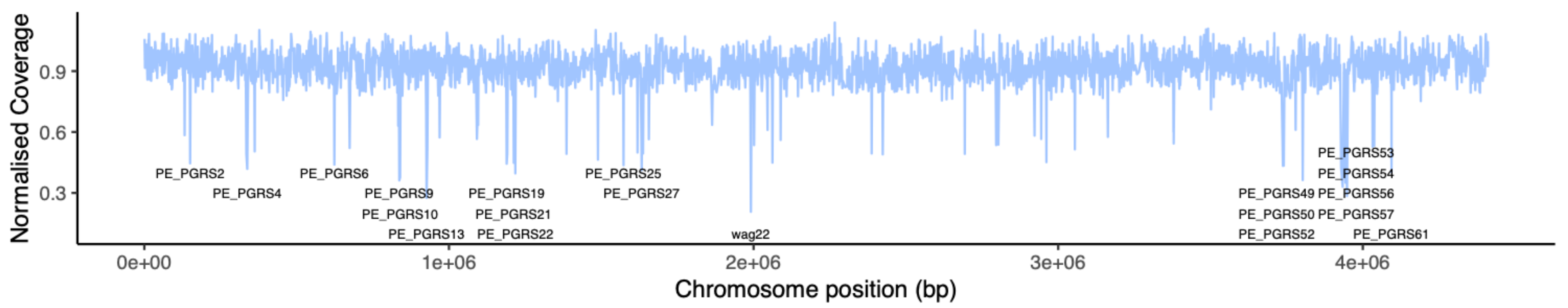

Normalised Coverage
0.9
0.6
0.3
PE_PGRS2
PE_PGRS4
PE_PGRS6
PE_PGRS9
PE_PGRS10
PE_PGRS13
PE_PGRS19
PE_PGRS21
PE_PGRS22
PE_PGRS25
PE_PGRS27
wag22
PE_PGRS49
PE_PGRS50
PE_PGRS52
PE_PGRS53
PE_PGRS54
PE_PGRS56
PE_PGRS57
PE_PGRS61
0e+00
1e+06
2e+06
3e+06
4e+06
Chromosome position (bp)

Figure S3. (A) Normalised coverage by gene category. The *pe*/*ppe* genes are divided in "Conserved" and "Structural Variant" based on the classification pipeline in **Figure S1**. Genes belonging to the "Unique *k-mer*" category are included in "Structural Variant". Every other gene in the genome is under "non-PE/PPE". Normalised coverage is shown by lineage for each category. Statistical differences were calculated between the means for each category. *** = P-value adjusted < 0.001

(B) Mean normalised coverage per gene along the genome. The 20 genes with the lowest mean normalised coverage are annotated.

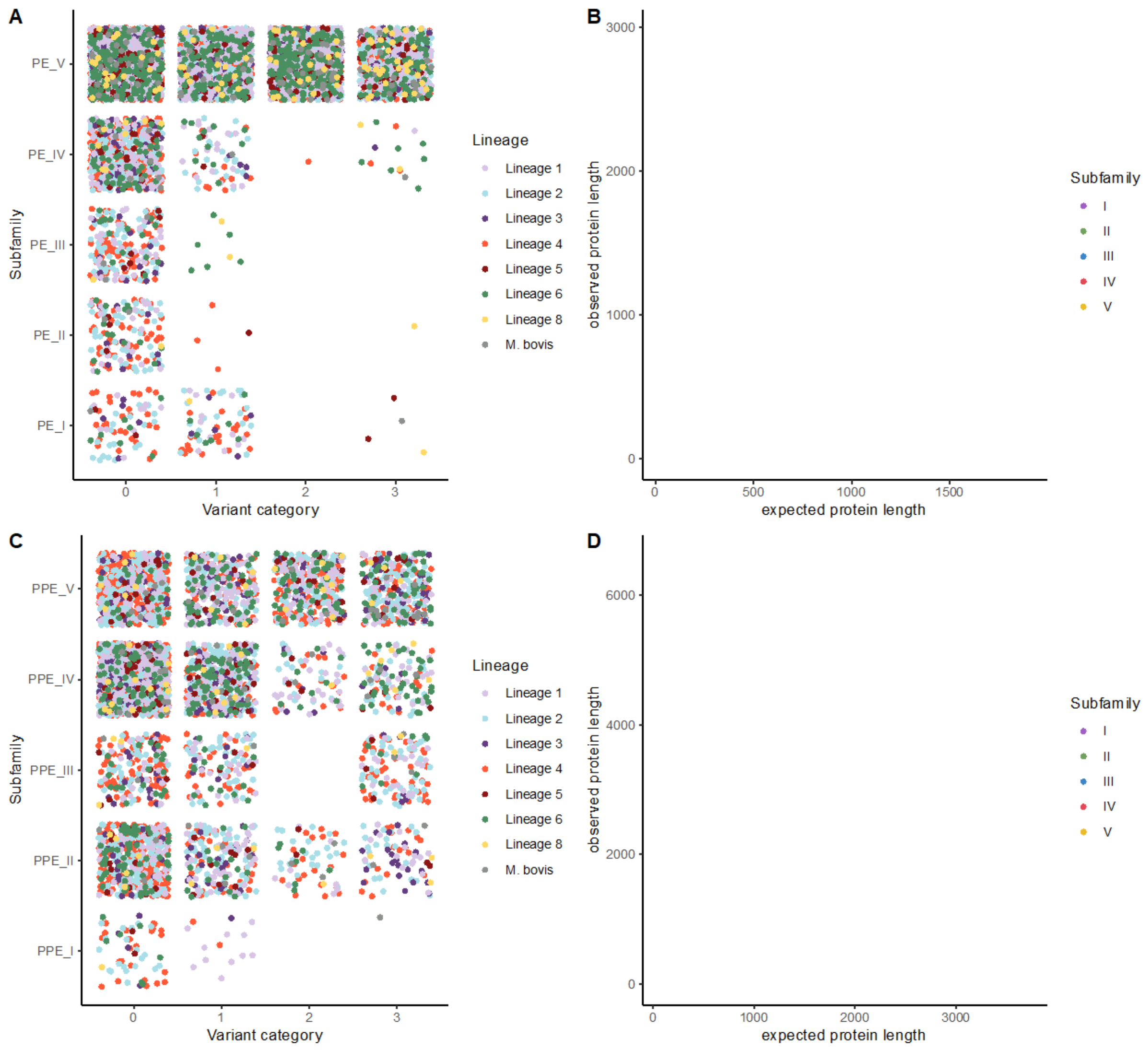

Figure S4. (A) and (C) Distribution of genes relative to their variant level (0 = no variant/synonymous SNPs; 1 = non-synonymous SNPs; 2 = in-frame indels; 3 = frameshift/premature stop codon/big deletion) and their sub-family, for *pe* family (A) and *ppe* family (C). (B) and (D) observed gene length vs expected gene length, coloured by sub-family, for *pe* family (B) and *ppe* family (D).

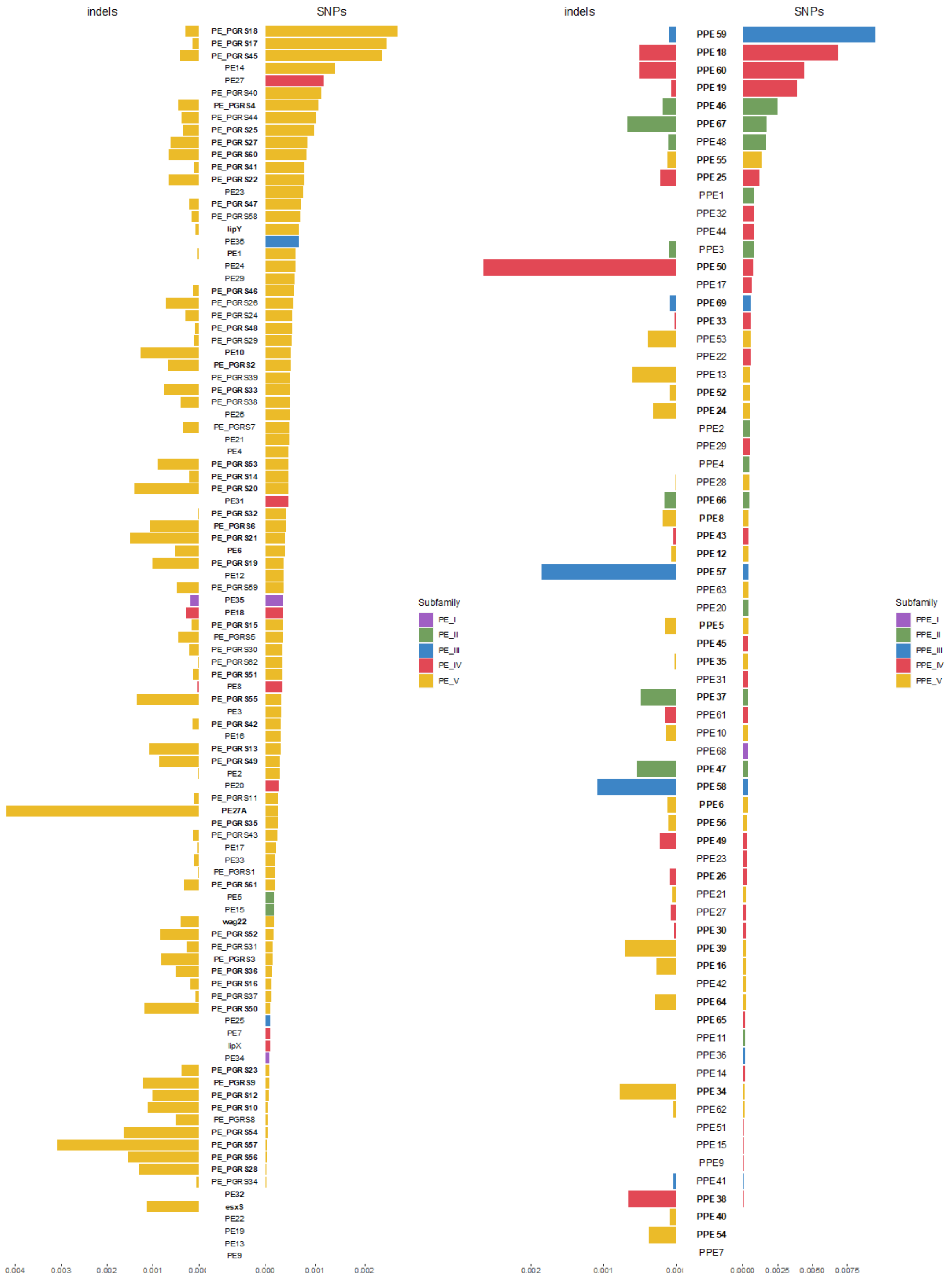

Figure S5. SNP and indel nucleotide diversity in both *pe* and *ppe* gene families. Colours correspond to subfamilies. Genes in bold belong to the class S or K.

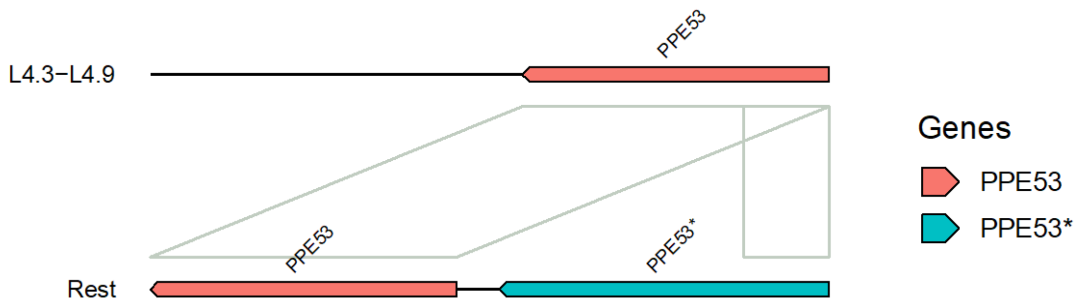

Figure S6. The *ppe53* locus representation in H37Rv and 4.3-4.9/8 lineage (first track) and the rest of lineages (second track). PPE53* indicates the 77% similar duplicated gene.

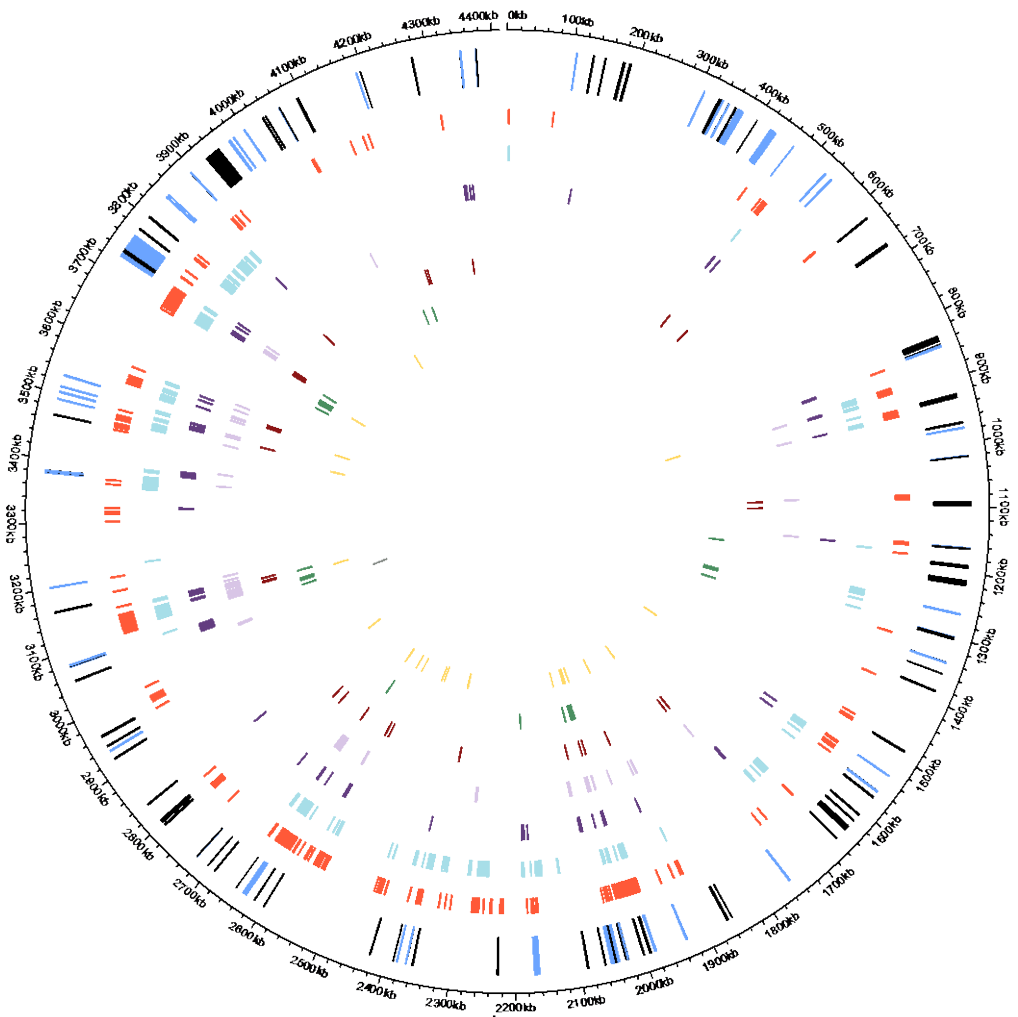

Figure S7. Circos plot showing the location IS*6110* along the genome for the different lineages. First track (outside to inside) refers to the location of *pe* (in black) and *ppe* (in blue) genes. Second to eighth track represent each of the position where IS*6110* is integrated in the samples belonging to each lineage as follows (in order): red for L4, light blue for L2, purple for L3, lilac for L1, brown for L5, green for L6, yellow for L8 and grey for *M. bovis* BCG.

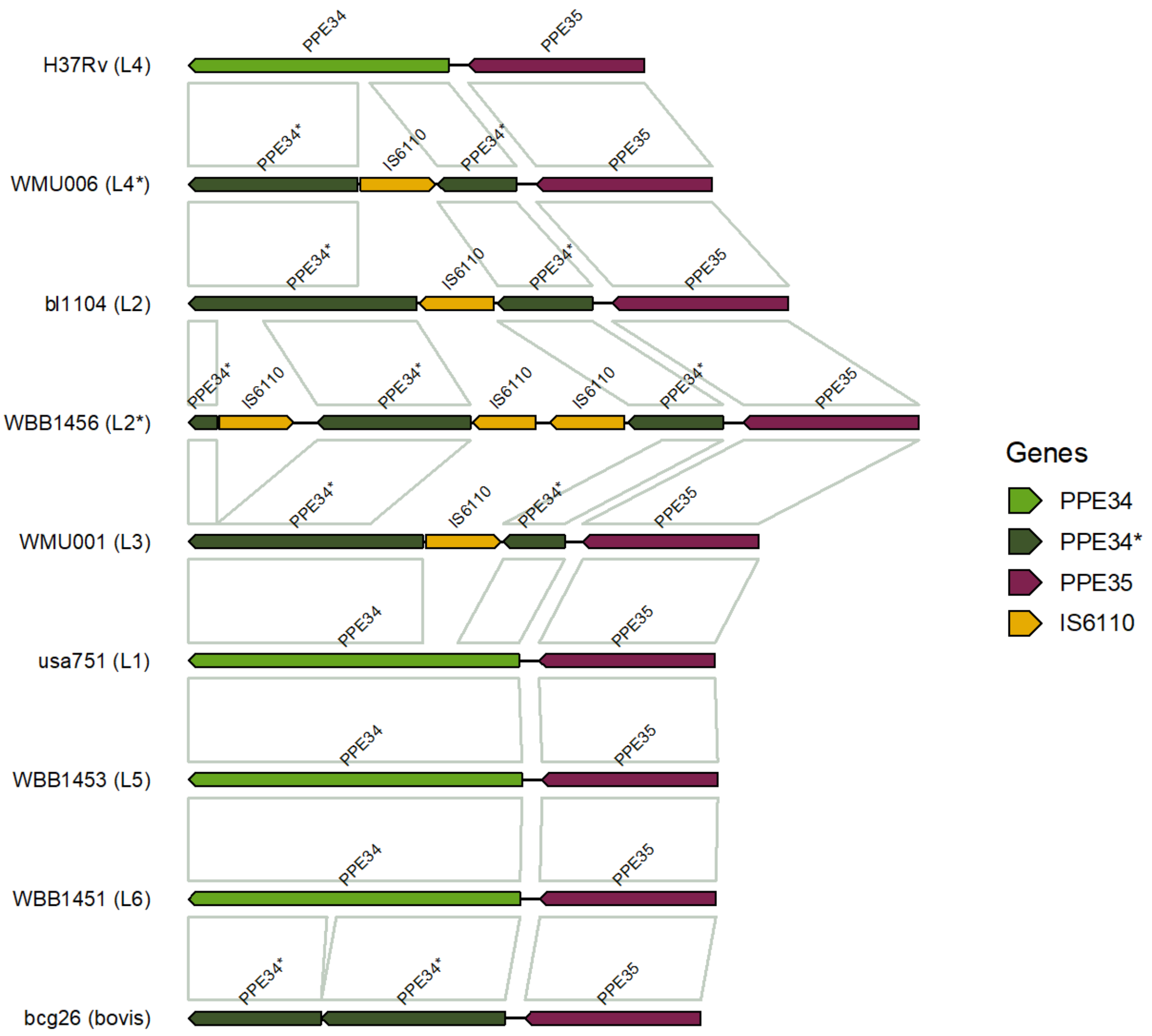

Figure S8. The *ppe34/35* loci organisation in representative strains for each lineage.

PPE34* = truncated *ppe34* gene

* Sporadic isolates

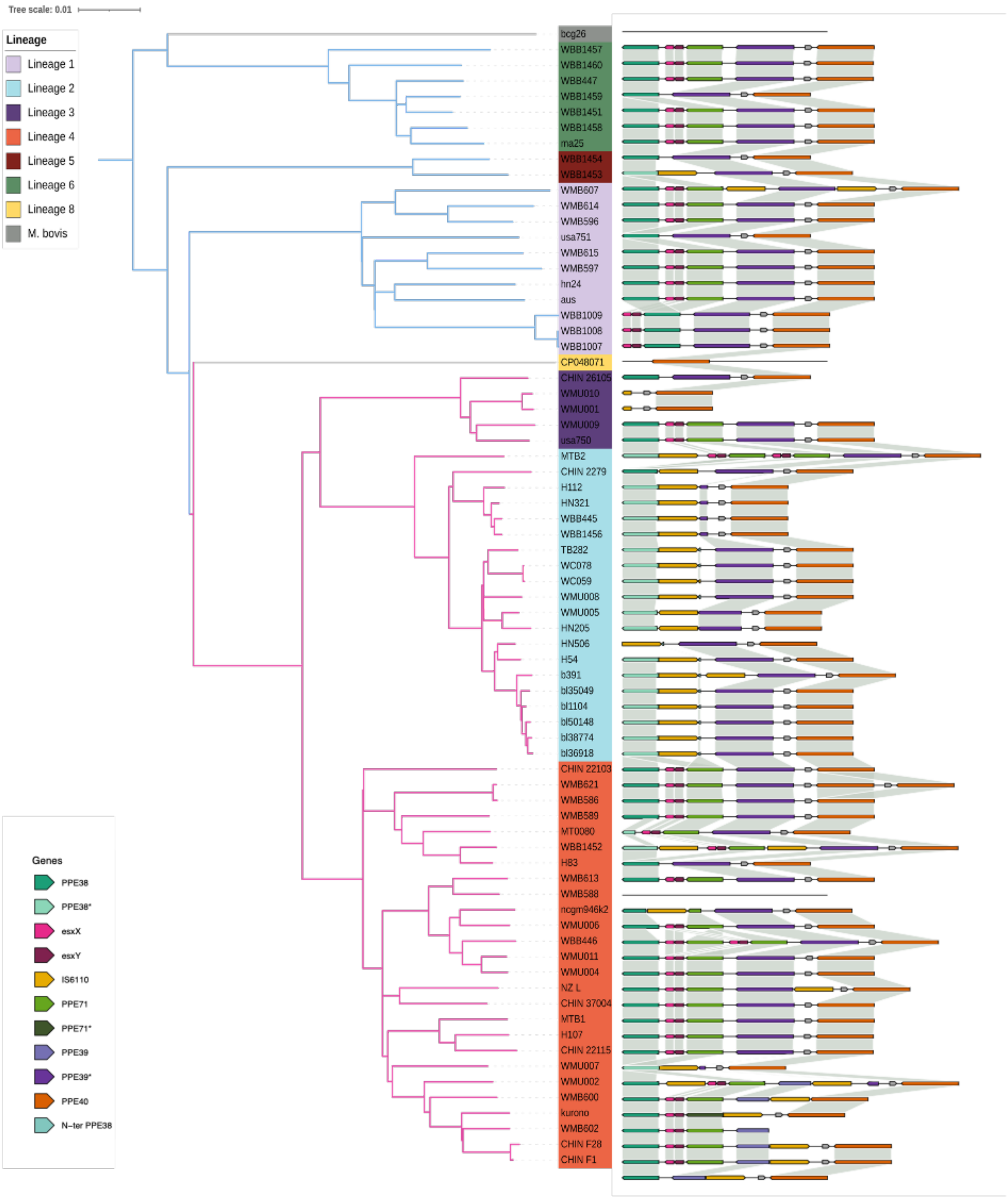

Figure S9. Configuration of *ppe38-ppe40* loci across the 73 samples analysed. H37Rv annotation shown at the bottom track. Samples ordered based on the phylogenetic tree shown on the left. Branches in pink represent modern lineages, in blue represent ancient lineages.

* Genes with different N-/C-terminal.

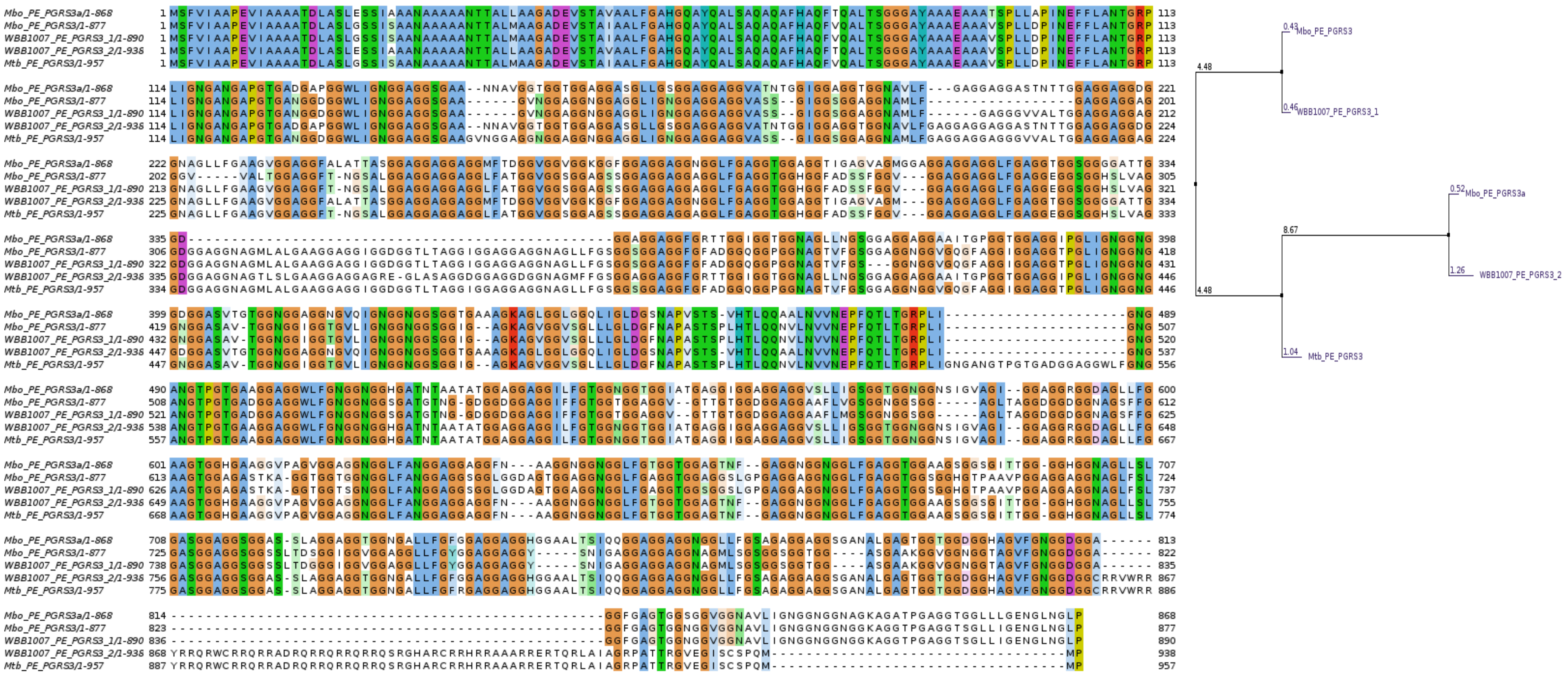

Figure S10. Protein sequence alignment of (from top to bottom) PE_PGRS3a from *M. bovis*, PE_PGRS3 from *M. bovis*, PE_PGRS3_1 from WBB1007 (*Mtb* L1), PE_PGRS3_2 from WBB1007 (*Mtb* L1) and PE_PGRS3 from H37Rv *Mtb*. Highlighted are the conserved residues across the different sequences. On the right, Neighbour joining tree using PID for the 5 sequences.

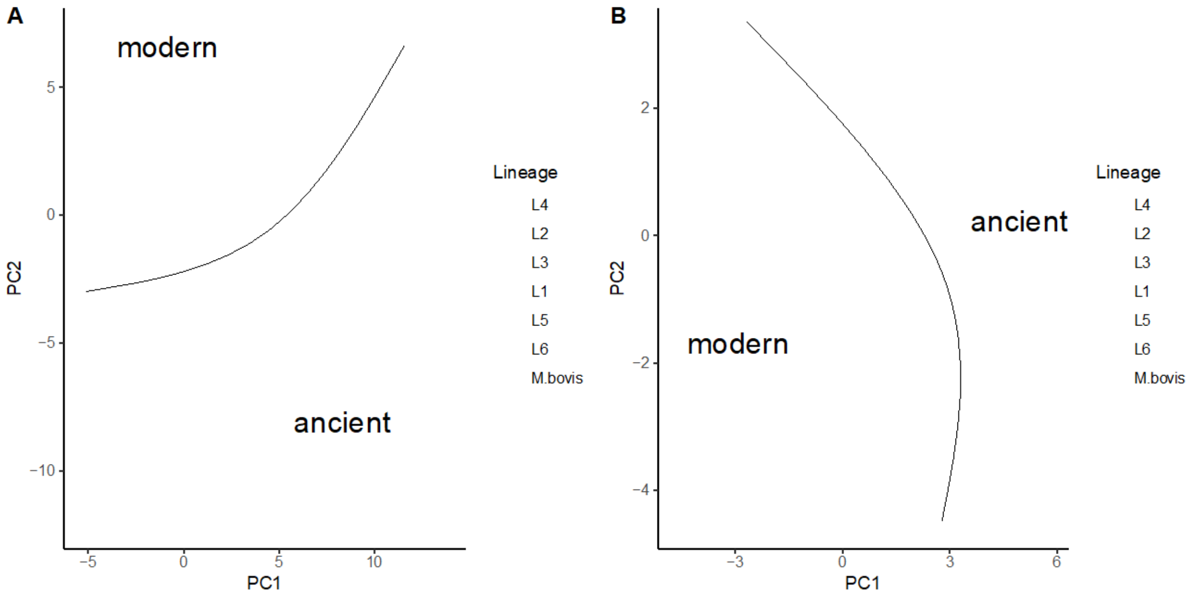

Figure S11. Principal component analysis (PCA) of SNPs (A) and indels (B) with samples coloured by lineage.

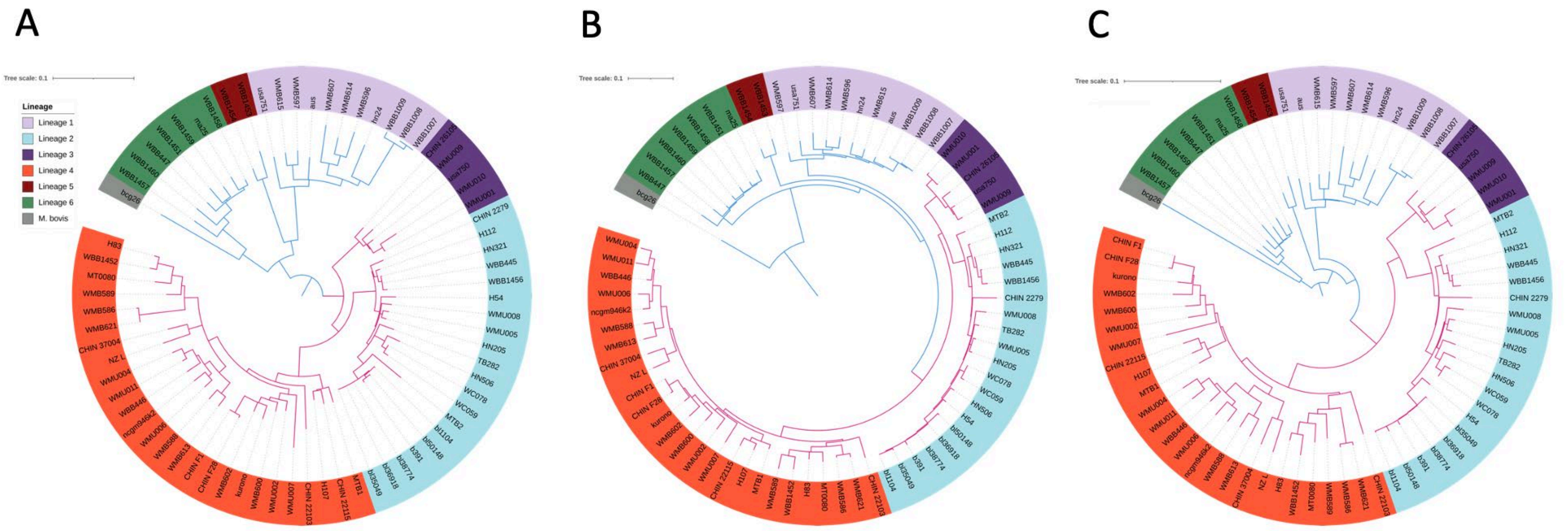

Figure S12. Maximum likelihood phylogenetic trees reconstructed with variants only in the *pe*/*ppe* genes as follows: (A) SNPs, (B) indels and (C) SNPs and indels.
